# Monitoring Twitter Conversations for Targeted Recruitment in Cancer Trials in Los Angeles County: Protocol for a Mixed-Methods Pilot Study

**DOI:** 10.2196/resprot.9762

**Published:** 2018-09-25

**Authors:** Katja Reuter, Praveen Angyan, NamQuyen Le, Alicia MacLennan, Sarah Cole, Ricky N Bluthenthal, Christianne J Lane, Anthony B El-Khoueiry, Thomas A Buchanan

**Affiliations:** 1 Institute for Health Promotion & Disease Prevention Research Department of Preventive Medicine, Keck School of Medicine University of Southern California Los Angeles, CA United States; 2 Southern California Clinical and Translational Science Institute Keck School of Medicine University of Southern California Los Angeles, CA United States; 3 USC Norris Comprehensive Cancer Center Keck School of Medicine University of Southern California Los Angeles, CA United States; 4 Division of Biostatistics Department of Preventive Medicine, Keck School of Medicine University of Southern California Los Angeles, CA United States; 5 Department of Medicine Keck School of Medicine University of Southern California Los Angeles, CA United States

**Keywords:** breast cancer, cancer, clinical research, clinical trial, colon cancer, kidney cancer, listening, lung cancer, lymphoma, monitoring, outreach, prostate cancer, recruitment, research participation, surveillance, Twitter, social media, social network

## Abstract

**Background:**

Insufficient recruitment of participants remains a critical roadblock to successful clinical research, particularly clinical trials. Social media provide new ways for connecting potential participants with research opportunities. Researchers suggest that the social network Twitter may serve as a rich avenue for exploring how patients communicate about their health issues and increasing enrollment in cancer clinical trials. However, there is a lack of evidence that Twitter offers practical utility and impact.

**Objective:**

This pilot study aimed to examine the feasibility and impact of using Twitter monitoring data (ie, user activity and their conversations about cancer-related conditions and concerns expressed by Twitter users in Los Angeles County) as a tool for enhancing clinical trial recruitment at a comprehensive cancer center.

**Methods:**

We will conduct a mixed-methods interrupted time series study design with a before-and-after social media recruitment intervention. On the basis of a preliminary analysis of eligible trials, we plan to onboard at least 84 clinical trials across 6 disease categories: breast cancer, colon cancer, kidney cancer, lymphoma, non-small cell lung cancer, and prostate cancer that are open to accrual at the University of Southern California (USC) Norris Comprehensive Cancer Center. We will monitor messages about these 6 cancer conditions posted by Twitter users in Los Angeles County. Recruitment for the trials will occur through the Twitter account (@USCTrials). Primary study outcomes—feasibility and acceptance of the social media intervention among targeted Twitter users and the study teams of the onboarded trials—will be assessed using qualitative interviews and the 4-point Likert scale and by calculating the proportion of targeted Twitter users who engaged with outreach messages. Second, impact of the social media intervention will be measured by calculating the proportion of enrollees in trials. The enrollment rate will be compared between the active intervention period and the prior 10 months as historical control for each disease trial group. This study has been funded by the National Center for Advancing Translational Science through a Clinical and Translational Science Award. Study approval was obtained from the clinical investigations committee at USC Norris and the institutional review board at USC.

**Results:**

Recruitment on Twitter started in February 2018. Data collection will be completed in November 2018.

**Conclusions:**

This pilot project will provide preliminary data and practical insight into the application of publicly available Twitter data to identify and recruit clinical trial participants across 6 cancer disease types. We will shed light on the acceptance of the social media intervention among Twitter users and study team members of the onboarded trials. If successful, the findings will inform a multisite randomized controlled trial to determine the efficacy of the social media intervention across different locations and populations.

**Trial Registration:**

ClinicalTrials.gov NCT03408561; https://clinicaltrials.gov/ct2/show/NCT03408561 (Archived by WebCite at http://www.webcitation.org/72LihauzW)

**Registered Report Identifier:**

RR1-10.2196/9762

## Introduction

### Background and Rationale

Recruitment of study participants in clinical research, particularly clinical trials, remains a critical roadblock to successful clinical research [1-5]. A recent systematic review found that 76.1% (131/172) of randomized clinical trials (RCTs) discontinued due to poor recruitment [6]. Insufficient and slow participant recruitment delays scientific and medical progress that could benefit patients and increases the financial costs to institutions, industry, and taxpayers [7-9]. According to the National Center for Advancing Translational Sciences (NCATS), “evidence-based strategies to trial participant recruitment and patient engagement” are required to address this challenge [10,11].

With billions of users, social media provides new venues to better connect potential participants with research opportunities in a variety of disease and health contexts [12,13]. The term *social media* describes widely accessible Web-based and mobile technologies that allow users to view, create, and share information and to participate in social networking [14,15]. Users can create a public or semipublic profile and maintain a list of other users they follow or with whom they may share content [16,17]. Nearly 70% of US adults use some type of social media, which varies by factors such as age, gender, race, and ethnicity across a range of social media such as Facebook, YouTube, Pinterest, Instagram, Twitter, LinkedIn, and Snapchat [18-20]. Social media provides an unprecedented opportunity for delivering information to reach large segments of the population [11] as well as hard-to-reach subpopulations that deal “with sensitive, stigmatizing, or rare health conditions” [13,21-24].

### Social Media Monitoring

The data that social media users generate by creating and interacting with Web-based information, also referred to as their *digital footprint* [25-28], provide a new data source for research. There has been an increase in using Twitter data for research, for example, to study public health and safety issues [29-33] and to monitor pharmaceutical products, potential drug interactions, and adverse events [34-38]. Social media monitoring (also referred to as *surveillance* or *listening*) describes the use of social media data (ie, user activity and their conversations) to gain insights into their interests, attitudes, and behaviors. In this study, we explore Twitter monitoring as a tool to examine cancer-related conditions and concerns expressed by Twitter users in Los Angeles County.

Using social media monitoring data for targeted clinical trial outreach is considered “active recruitment that occurs when research staff members approach and interact with specific individuals with the aim of enrolling them in research, usually on the basis of knowledge of characteristics that would make them suitable candidates for particular trials” [39]. To date, the clinical research community has focused little attention on the use of social media data in clinical research recruitment, for example, to identify potential study participants who have expressed specific health conditions and concerns and are most likely to participate in a clinical study [40,41]. Furthermore, sponsors have reported the lack of experienced vendors and internal teams as well as clinical research offices as main barriers to the adoption of social media monitoring and outreach strategies [42].

### Twitter

Nearly 25% of US adults use the social media platform Twitter with billions of users across the world [18,19]. Twitter allows users to post short messages (tweets) that are limited to 280 characters [43]. Users can search for any public message and further engage with tweets, that is, they can *like*, reply, and *retweet* (share) them. Twitter is primarily a public social network. By default, basic Twitter account information such as the profile name, description, and location is public unless a user decides to opt out and make a private account [44,45].

### Twitter and Cancer Communication

Due to the more public nature of Twitter, previous research suggested that Twitter provides a “rich and promising avenue for exploring how patients conceptualize and communicate about their specific health issues” [46]. The increasing use of Twitter among members of the cancer disease community is evidenced by the abundance of cancer-related hashtags used by Twitter users in their messages [46-51]. A hashtag is a user-generated word or phrase preceded by a hash or pound sign (#) and used to identify messages on a specific topic on Twitter. For example, among the most widely recognized hashtags used in Twitter messages for breast cancer are #breastcancer, #bcancer, and #BCSM (breast cancer and social media) [52]. Researchers also emphasized Twitter as a “powerful and important tool in implementing and disseminating critical messages to the community in real-time” [53,54] and a “way to communicate with the public about cancer clinical trials and increase awareness and enrollment” [55]. A study on lung cancer, for example, found that Twitter messages focused on support, prevention, and clinical trials and were predominantly authored by individuals [55]. However, there is a lack of evidence that Twitter offers practical utility and impact.

### Social Media and Clinical Trial Recruitment

More investigators are incorporating social media in their study recruitment strategy for human subjects’ research in general and clinical trials with varying results [56-66]. In some cases, they also compared the social media recruitment outcomes to traditional methods [13]. However, the development of evidence-based social media recruitment methods based on the existing data poses challenges and requires more consistent and transparent frameworks for data collection, study design, quality assessment, debiasing techniques for social media data, and systematic reporting standards and clearly defined metrics [67-70]—most of which are currently lacking. In fact, many RCTs published in major journals do not provide adequate information about the patient recruitment process, including how they incorporated and measured social media [71]. As a result, it is difficult to gauge the effectiveness of social media–driven recruitment methods and their cost effectiveness across different disease types, target populations, and social media platforms.

### Study Objective and Hypothesis

The objective of this pilot study is to examine the feasibility and impact of using targeted Twitter monitoring as a tool for enhancing and complementing clinical trial recruitment among Twitter users in Los Angeles County at a comprehensive cancer center. In collaboration with the USC Norris Comprehensive Cancer Center (USC Norris) at the University of Southern California (USC), where the study will be implemented, we will conduct a mixed-methods interrupted time series study design with a before-and-after social media recruitment intervention. We plan to onboard all clinical trials open for accrual, at least 84 based on a preliminary analysis of eligible trials for this study in 6 cancer disease categories: breast cancer, colon cancer, kidney cancer, lymphoma, non-small cell lung cancer, and prostate cancer (Multimedia Appendix 1, page 24). We will monitor messages about these cancer conditions posted by Twitter users in Los Angeles County. We hypothesize that Twitter monitoring data serve as a useful tool to enhance and complement clinical trial recruitment efforts, more specifically to identify and recruit participants for cancer trials, which may vary in success based on the cancer disease type, disease-related issues that impact trial eligibility, and other demographic factors.

The study has 2 primary outcomes. First, the feasibility and acceptance of the social media intervention among targeted Twitter users and the study teams of the onboarded trials, which will be assessed through qualitative interviews using a 4-point Likert scale and a number of quantitative measures to calculate the proportion of targeted Twitter users who engaged with outreach messages (measured through Twitter replies, mentions, *likes*, *retweets*, direct messages, following, and contact form use on the trial webpage). Second, the impact of the social media intervention will be measured by calculating the proportion of people who consented and enrolled in trials (ie, enrollment rate). The enrollment rate will be compared between the active intervention period and the prior 10 months as historical control for each disease trial group. To aid in the design of larger and more definitive studies, we also intend to estimate the effect size of the number of people enrolled associated with the use of targeted social media monitoring on Twitter as a tool for enhancing cancer trial recruitment. Finally, we aim to establish a method for implementing a social media–driven centralized clinical trial recruitment approach at a comprehensive cancer center, taking into account their internal workflows and processes. Textbox 1 lists the specific research questions we intend to answer with this study.

This protocol paper provides a detailed description of a social media monitoring and recruitment intervention on Twitter as well as clear metrics to assess its feasibility and impact. Such metrics include data on eligible Twitter users in Los Angeles County who have expressed specific health conditions and concerns; outreach messages to targeted Twitter users; their engagement with these messages either via Twitter or the trial webpage, completion of prescreening and screening procedures, and final consent and enrollment. It is our goal to contribute to the development of more transparent, evidence-based social media recruitment methods and measurement frameworks. Our findings will provide pilot data on the use of Twitter as a resource for identifying and recruiting clinical trial participants across 6 different cancer disease types and help to explore a new path for the application of publicly available Twitter data in support of centralized trial recruitment at a comprehensive cancer center.

**Research questions we intend to answer with this study.**

1. How feasible is the application of social media monitoring to enhance recruitment in cancer trials among Twitter users in Los Angeles County?
  - What are the reasons for not enrolling eligible clinical trials?
  - How many trials and disease categories can be monitored for (on Twitter) at a time?
  - How does the proposed social media monitoring and recruitment intervention affect the workflow of the study team?
  - How much time and effort does it take to respond to the resulting inquiries from Twitter, to decide whether or not to follow up with a potential participant, and to bring the patient in for an evaluation?
  - How many targeted Twitter users engaged with the outreach message (measured through Twitter replies, mentions, likes, retweets, direct messages, following, and contact form use on the trial webpage)?
  - How does the social media intervention affect potential participants’ satisfaction and their level of privacy concern?
  - How many targeted Twitter users were prescreened for eligibility?
  - How many targeted Twitter users were eligible based on prescreening?
  - How many targeted Twitter users were screened for eligibility?
  - How many targeted Twitter users were eligible based on screening?
  - How diverse are Twitter users that were targeted for outreach?
  - How diverse are Twitter users that were prescreened?
  - How diverse are Twitter users that were eligible based on prescreening?
  - How diverse are enrolled trial participants (measured by age, gender, and racial and ethnic background)?
2. How effective is the application of social media monitoring to enhance enrollment for clinical trials?
  - What is the enrollment rate (ie, number of people who consented and enrolled divided by the total number of people contacted) that results from social media monitoring on Twitter (recruitment rates will be compared between the active intervention period and the prior 10 months as historical control for each disease trial group)?

## Methods

### Ethical Approval and Protocol Amendments

Study approval was obtained from the clinical investigations committee (CIC) at USC Norris (Protocol 0S-17-7; Multimedia Appendix 2) and the institutional review board (IRB) at USC (Protocol HS-17-00811; Multimedia Appendix 3). This study is also registered at ClinicalTrials.gov (NCT03408561) [72]. Any amendments made to the study protocol will be reported to the IRB at USC and the Clinical Investigation Support Office (CISO) at USC Norris.

### General Study Design and Study Setting

We will use a mixed-methods interrupted time series study design with a before-and-after social media intervention, also including qualitative interviews using a 4-point Likert scale, that will be implemented at USC Norris. Using both qualitative and quantitative analyses can enhance the validity of study findings [73,74]. The National Cancer Institute NCI has designated USC Norris as one of the nation's comprehensive cancer centers, a select group of institutions providing leadership in cancer treatment, research, prevention, and education. Data analysis and all other matters related to drafting of the manuscript will occur at the School of Medicine of USC.

### Intervention

The social media monitoring and recruitment intervention to be tested in this study involves 2 steps: (1) monitoring disease-specific conversations by Twitter users in Los Angeles County with a focus on 6 cancer topics: breast cancer, colon cancer, kidney cancer, lymphoma, non-small cell lung cancer, and prostate cancer (Textbox 2), and (2) contacting eligible Twitter users (Textbox 3) via public reply with information about disease-specific cancer trials that are open to accrual at USC Norris.

To access public Twitter user data, we will use Symplur Signals [75], a health care social media analytics platform that maintains a database of curated disease- and health-related Twitter conversations and user data, updated daily and easily sortable by social media user type (eg, patient, physician, and health care organization), location and time zone, language, disease or health interests, and Twitter message content. We will review both retrospective and prospective data published by Twitter users in Los Angeles County between July 28, 2017 and November 30, 2018 to identify potential trial participants for each trial disease group. Two independent coders (including the study co-principal investigator, co-PI) will review the Twitter data to identify Twitter users eligible for targeted outreach.

### Randomization

Several aspects of this study will be randomized to reduce selection bias. First, the order in which the cancer trial disease groups will be onboarded in this study will be shuffled randomly using a Fisher-Yates shuffle [76]. Second, the selection of the initial outreach messages will be randomized using a *true* random number generator [77]. Third, those Twitter users who are eligible and consent to enroll in one of the cancer trials will be randomized if required by the individual trial.

**Study eligibility criteria for clinical trials.**

Inclusion criteria

- Focus on one of the 6 cancer disease types: breast cancer, colon cancer, kidney cancer, lymphoma, non-small cell lung cancer, or prostate cancer
- Be institutional review board–approved and open to accrual at the USC Norris Comprehensive Cancer Center
- Be a phase 1 trial in expansion, phase 2 or 3
- Be an interventional trial
- Recruit in English
- Recruit for at least 9 months at the point of enrollment
- Set a monthly accrual target ≥1 and annual accrual target ≥12

Exclusion criteria

- Phase 1 trials in dose escalation

**Study eligibility criteria used for Twitter user outreach.**

Inclusion criteria

- Be located in Los Angeles County based on the self-reported description provided on user’s Twitter profile (Multimedia Appendix 4)
- Mention in any of their Twitter messages at least one word or hashtag related to the 6 cancer disease types (Multimedia Appendix 5)
- Message is an original Twitter message or reply to another user’s message
- Message indicates that Twitter user has been diagnosed with the cancer disease or that they know someone who has been diagnosed with the cancer disease

Exclusion criteria

- Cancer patients in remission (ie, signs and symptoms of that cancer have reduced)
- Cancer survivors (ie, there are no traces of cancer left)
- Persons younger than 18 years
- Persons who note that a relative or friend has died of the disease
- Retweets (ie, user shares message other Twitter users sent)

### Eligibility

#### Characteristics of Eligible Clinical Trials

Clinical trials will be required to meet the eligibility criteria outlined in Textbox 2. The trial selection is independent of the stage of disease. The 6 cancer trial disease categories were selected based on 2 factors: the results of a preliminary Twitter data analysis in California to determine the most frequently mentioned cancer topics in the region, and the number of clinical trials at USC Norris that are open for accrual. Between January 1, 2016 and January 30, 2017, we found 36,502 Twitter users in California who had sent a total of 159,396 Twitter messages in English including at least one of the selected 6 cancer disease terms (unpublished data from Symplur Signals). Additionally, a preliminary analysis of clinical trials at USC Norris between January 1, 2017 and July 7, 2017 identified 84 clinical trials that were open for accrual and would be eligible for this study (Multimedia Appendix 1, page 24). We intend to onboard all eligible trials in the select 6 cancer disease areas that are open for accrual at the time of the onset of this study. Social media monitoring on Twitter will be used to identify potential cancer trial participants for all onboarded trials. We refer to this approach as “centralized trial recruitment” because we cluster trials into groups by disease and promote only 6 disease trial groups on Twitter rather than each individual trial. Including all cancer trials related to one disease type aligns with the Center’s internal screening and triage process. The physicians and clinical research coordinators are divided into disease-specific teams and therefore will consider potential trial participants for all the relevant trials in that disease area. Finally, to reduce selection bias, we will onboard one disease trial group every 2 weeks in a randomized order. Once a clinical trial disease group is onboarded, the trials in that group stay on for the period of this study.

#### Characteristics of Eligible Twitter Users Selected for Targeted Outreach

**Figure 1:**
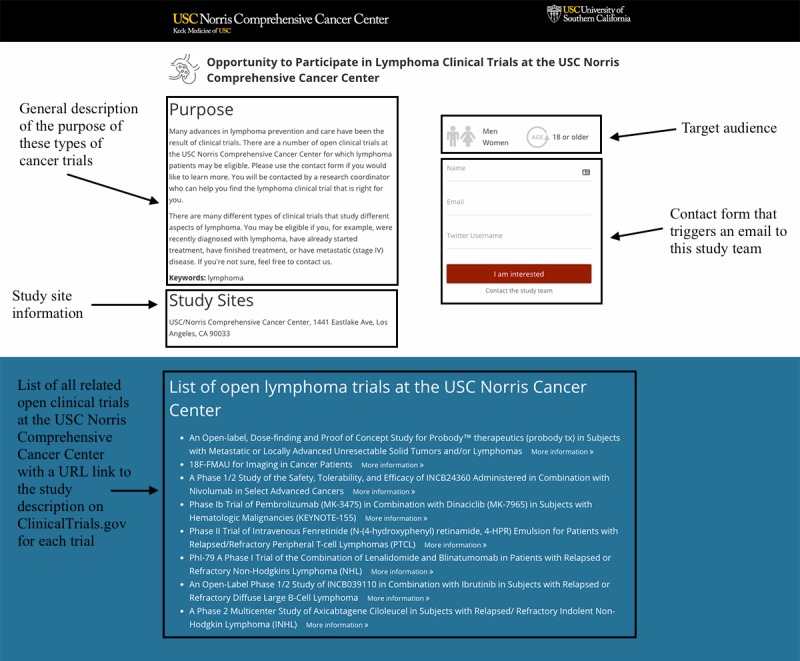
Example of a webpage (part 1) that includes information about the clinical trials on lung cancer that are open to accrual at the USC Norris Comprehensive Cancer Center (USC Norris). Squares and numbers show the following page elements: (1) the general description of the purpose of these types of cancer trials; (2) the study sites; (3) the target recruitment population; (4) a contact form that triggers an email to this study team; and (5) a list of the clinical trials at USC Norris including a URL link to the description on ClinicalTrials.gov for each trial.

**Figure 2:**
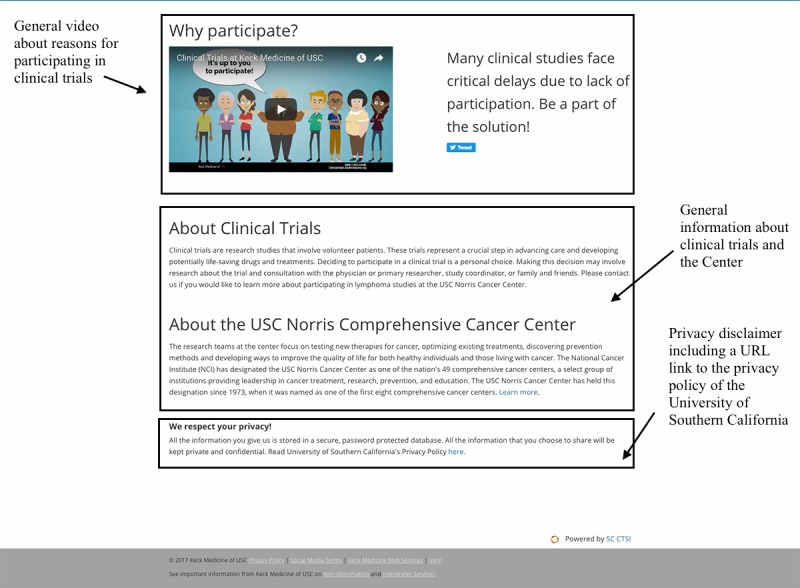
Example of a webpage (part 2) that includes information about the clinical trials on lung cancer that are open to accrual at the USC Norris Comprehensive Cancer Center (USC Norris). Squares highlight the following page elements: a general video about reasons for participating in clinical trials; general information about clinical trials and USC Norris; and a privacy disclaimer including a URL link to the privacy policy of the University of Southern California.

**Figure 3:**
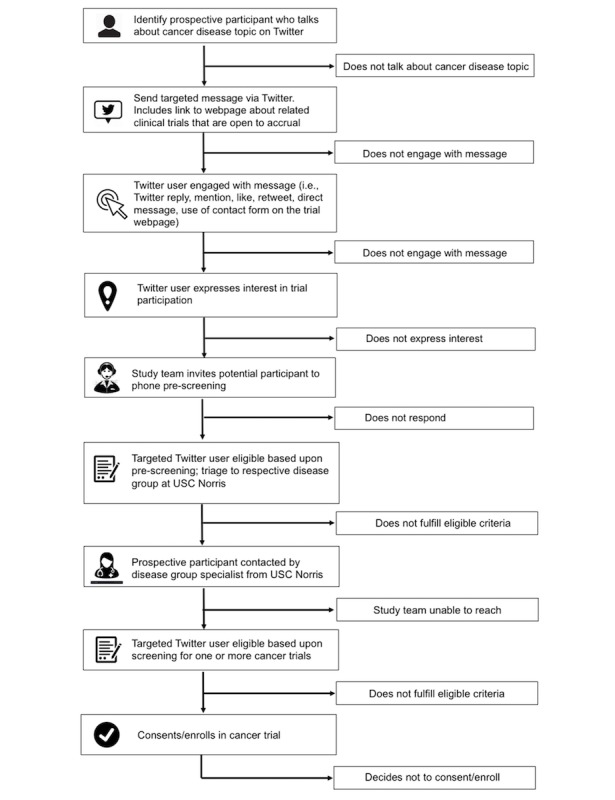
Study flow diagram of study design and procedures. USC Norris: USC Norris Comprehensive Cancer Center.

Participant recruitment for the onboarded clinical trials will occur on the social network Twitter. The study will be limited to those Twitter users who meet the eligibility criteria outlined in Textbox 3. We will apply both Boolean and Regex location code categories (Multimedia Appendix 4) to determine user locations. Any Twitter user located in Los Angeles County who mentions one or more words related to the selected cancer disease topics (Multimedia Appendix 5) will be contacted via Twitter using the public reply feature. We will include all potential trial participants in this study who express interest in trial participation via Twitter or through the contact form on the trial webpage (Figures 1 and 2) in response of the targeted outreach. They will be invited to an initial phone prescreening. See Figure 3 for details on study design and procedures.

### Consent, Prescreening, and Screening Procedures

Prescreening of Twitter users to determine if they should be triaged to the USC Norris team will occur over the phone. See Multimedia Appendix 6 for the complete prescreening questionnaire. Verbal consent to participate in the social media study will be obtained before the initial prescreening. Persons who are eligible for triage to USC Norris must meet the eligibility criteria outlined in Textbox 4. After triage to the respective cancer disease contact at USC Norris, a physician and/or clinical research coordinator will contact the potential participant to obtain additional clinical information, describe available trials, and arrange an in-person evaluation to determine the eligibility for one of the individual trials, if appropriate. After the in-person visit, if the patient is considered to be a potential candidate, the physician will complete the informed consent process with the participant for the specific trial in question, and the formal screening and eligibility work-up will be completed. Twitter users who do not meet the eligibility criteria of any of the cancer trials open to accrual will be excluded from participation in this study, as well as persons who may be eligible (eg, disease, histology, stage, and prior treatment) but do not meet additional trial-specific requirements such as insurance or allergy to drug. These may vary by clinical trial. We will count these people as engaged but not enrolled and document the specific reasons.

### Recruitment

#### Onboarding of Clinical Trials

Study teams of the onboarded cancer trials will not receive monetary or any other compensation for enrolling their clinical trials in this study. We will work closely with the CISO team at USC Norris to recruit all clinical trials in the select cancer disease areas that are open for accrual.

#### Recruitment of Twitter Users in Los Angeles County

Participants (ie, targeted Twitter users in Los Angeles County) will not receive monetary compensation for participating in the social media study but may receive compensation if they consent to participate in one of the clinical trials depending on the trial. Participants will be recruited using public replies to their Twitter messages that mention words related to the selected cancer disease types (Multimedia Appendix 5). We will use the @USCTrials Twitter account [78] for sending the targeted outreach messages. We will not use any advertised (paid) messages because Twitter does not permit paid advertisement of clinical trials [79].

The outreach (recruitment) messaging consist of three types of messages, which we refer to as the “outreach message package” (Textbox 5). An initial outreach message (Textbox 5), which is selected randomly (see Randomization section), is a personalized message to the person using their name (if available on Twitter) or Twitter handle (eg, @JaneDoe) referring to their previous mention of a specific cancer disease condition or concern and offering them more information (eg, “Dear Michael: We noticed your interest in #lungcancer and wanted to share the latest open clinical research opportunities @KeckMedUSC. You can find more information here: [URL] #ClinicalTrial”). The second message introduces the research project ensuring *investigator transparency* that “demands investigator truthfulness and honesty when interacting with research volunteers” and promoting “public trust in the research enterprise” (Textbox 5) as suggested by Gelinas et al [39]. The third message includes a disclaimer about the privacy risks of Twitter using the Privacy by Design framework, a globally recognized standard for privacy protection as suggested by Bender et al [24]. The disclaimer message points out security as a possible threat to privacy of social media users if the data are leaked (Textbox 5). Via the URL link in each message, Twitter users interested in the respective cancer clinical trials will be directed to a webpage (Figures 1 and 2) that includes information about *all* clinical trials in this disease category that are open to accrual at USC Norris. We call this approach *centralized trial recruitment*. Including all cancer trials related to one disease type in the same webpage aligns with the center’s internal screening and triage process; the physicians and clinical research coordinators are divided into disease-specific teams and therefore will consider potential trial participants for all the relevant trials in that disease area.

**Study eligibility criteria for triage to the respective cancer disease contact at the USC Norris Comprehensive Cancer Center.**

Inclusion criteria

- Have active cancer or recently underwent surgical resection for cancer
- Cancer is visible on scans (computed tomography, magnetic resonance imaging) unless recently resected
- Able to do activities of daily life independently (eg, eating, drinking, and bathing)

Exclusion criteria

- Have completed curative cancer therapy more than 12 months ago

**Outreach message package used to contact prospective clinical trial participants on Twitter.**

Initial outreach messages (random selection for each outreach: the parameter “#disease” will be replaced with the respective cancer disease type [ie, breast cancer, colon cancer, kidney cancer, lymphoma, non-small cell lung cancer, and prostate cancer] and the parameter “URL” with a shortened link to the related trial disease group webpage):

- We noticed your interest in #disease and wanted to share the latest open clinical research opportunities @KeckMedUSC. You can find more information here: URL #ClinicalTrial
- We noticed your mention of #disease and wanted to reach out. Did you know about these open #disease studies @KeckMedUSC? You can find more information here: URL #ClinicalTrial
- We noticed your interest in #disease and wanted to share the latest open clinical research opportunities @KeckMedUSC. You can find more information here: URL #ClinicalTrial
- We noticed your interest in #disease and wanted to share that the following #disease clinical trials @KeckMedUSC are looking for participants. More information is available here: URL #ClinicalTrial
- We noticed your interest in #disease and thought you might be interested in open #disease clinical trials @KeckMedUSC that are looking for participants. More information is available here: URL #ClinicalTrial

Project-related message: We’re reaching out to you as part of a research project trying to understand if Twitter can be used to better connect patients with clinical research opportunities.

Privacy and security disclaimer: The security of social media is not guaranteed. Contact us about the study. Don’t post if concerned about privacy.

The trial disease group webpage (Figures 1 and 2) includes a general description of the purpose of these types of cancer trials, the target recruitment population, study site information, a contact form that triggers an email to this study team, a list of the clinical trials at USC Norris including a URL link to the description on ClinicalTrials.gov for each trial, a general video about reasons for participating in clinical trials, general information about clinical trials and USC Norris, and a privacy disclaimer including a URL link to the privacy policy of USC. Twitter users will also be able to contact the study team through Twitter using either the public reply or mention options or the direct message feature that allows them to send private messages to the @USCTrials Twitter account. As part of the outreach and recruitment approach, we (ie, @USCTrials Twitter account) will also *follow* each targeted Twitter user to whom the outreach message package was sent. This adds the respective person (Twitter account) to the network of @USCTrials and allows them to send us private, direct messages to the @USCTrials account, which some Twitter users may prefer. By default, Twitter only allows direct messages to be sent to followers to prevent misuse.

#### Recruitment of Clinical Trial Study Team Members

Study teams of the onboarded cancer trials will not receive monetary or any other compensation for participating in the study team interviews. We will work closely with the CISO team at USC Norris to invite and recruit (via email using USC’s email system) study team members of the enrolled clinical trials to participate in an interview (ie, PIs, clinical research coordinators, and recruitment specialists).

### Qualitative Interviews

#### Prescreening Interviews With Twitter Users

Brief survey interviews with targeted Twitter users (ie, potential study participants who contacted the study team in response to the social media outreach) will be conducted by the study team during prescreening over the phone. The goal of the prescreening questionnaire (Multimedia Appendix 6) is to collect demographic information about the Twitter users who expressed interest in trial participation, to better understand their perception of the social media intervention, in particular, their level of privacy concern, and to determine their eligibility regarding the triage to the USC Norris team for further screening.

#### Postqualitative Interviews With Clinical Trial Study Team Members

Postqualitative interviews with study team members of the onboarded clinical trials (PIs, clinical research coordinators, and recruitment specialists) will be undertaken to explore their views of and acceptance of the social media intervention. The interview guide will be based on the research questions to assess feasibility and acceptance (Textbox 1). The interview guide is under development and will be submitted to the USC IRB for review. As data are collected and the study team conducts the initial analyses, elements of the guide may require revision, and any important issues that emerge will be added. Interviews will be audio-recorded and take approximately 1 hour.

#### Outcomes

The study has 2 primary outcomes. The first outcome will be feasibility and acceptance of the social media intervention among targeted Twitter users and the study teams of the onboarded trials. We will conduct qualitative interviews using a 4-point Likert scale to assess the feasibility and the level of acceptance. We will further use a number of quantitative measures to assess the feasibility by calculating the proportion of targeted Twitter users who engaged with outreach messages (measured through Twitter replies, mentions, *likes*, *retweets*, direct messages, following, and contact form use on the trial webpage). The second outcome will be the impact of the social media intervention, which we will measure by calculating the proportion of people who consented and enrolled in trials (ie, enrollment rate). The enrollment rate will be compared between the active intervention period and the prior 10 months as historical control for each disease trial group (see Textbox 6 for a list of primary outcomes and definitions to be included in this study).

#### Control Group

There is no prospective control group in this study. Recruitment rates that result from the social media intervention will be compared between the active intervention period and the prior 10 months as historical control for each disease trial group.

#### Sampling Quota

Due to the lack of previous studies that explored this type of social media monitoring intervention for clinical trial recruitment, we do not provide a sampling quota. That said, we looked at research studies that had recruited participants via Twitter to determine potential baseline data or estimates. However, as the scoping review by Topolovec-Vranic and Natarajan from 2016 demonstrates [13], there are multiple issues regarding the comparison of social media recruitment strategies and results used in different studies. The authors looked at 30 research studies that had used social media for recruitment and compared the results with at least one traditional recruitment method. The review shows the lack of reporting standards for social media recruitment. Among the issues, study teams report combined data for social media recruitment (eg, for Facebook and Twitter combined) so that it is impossible to know how many participants were recruited using one social media type. The definition of social media used by authors also varies across the literature. Some study teams combine websites such as Twitter and Facebook with other types of tools such as Craigslist and classify all of them as social media. This has an effect on the results and conclusions that can be drawn about their effectiveness and enrollment rates.

**Primary outcomes and definitions.**

Outcome: Feasibility and Acceptance

- Reasons for not enrolling in eligible clinical trials
- Number of cancer disease types and studies that can be monitored on Twitter simultaneously by the study team
- Effect of social media intervention on workflow of study teams
- Time and effort required to respond to the resulting inquiries by targeted Twitter users (eg, decide whether or not to follow up with a potential participant, to bring the patient in for an evaluation)
- Diversity of Twitter users targeted for outreach (measured by age, gender, and racial and ethnic background)
- Number of targeted Twitter users who engaged with outreach message (measured through Twitter replies, mentions, likes, retweets, direct messages, following, and contact form use on the trial webpage)
- Effect of social media intervention on potential participants’ satisfaction and their level of privacy concern
- Number of targeted Twitter users who were prescreened for eligibility
- Diversity of prescreened Twitter users (measured by age, gender, and racial and ethnic background)
- Number of targeted Twitter users who were eligible based on prescreening
- Diversity of Twitter users who were eligible based on prescreening (measured by age, gender, and racial and ethnic background)
- Number of targeted Twitter users who were screened for eligibility
- Number of targeted Twitter users who were eligible based on screening
- Diversity of Twitter users who were eligible based on screening (measured by age, gender, and racial and ethnic background)
- Diversity of enrolled participants (measured by age, gender, and racial and ethnic background)

Outcome: Impact

- Enrollment rate per month: number of people enrolled per month divided by number of people targeted on Twitter per trial disease group per month (recruitment rates that result from the social media intervention will be compared between the active intervention period and the prior 10 months as historical control for each disease trial group)

Finally, there are few research studies that report the use of Twitter for participant recruitment. However, these studies focused on other diseases or health conditions (eg, pregnancy, smoking cessation). We decided not to use the recruitment results reported by these studies as baseline data or estimates, as we believe that the disease or health condition of a study as well as the type of “ask” (eg, completion of a Web-based survey, participation in clinical trial) influences the engagement and enrollment rate among potential participants. Hence, we will use the preliminary data from this study to estimate the effect size of the number of people enrolled associated with the use of targeted social media monitoring on Twitter as a tool for enhancing recruitment to cancer trials.

### Data Collection, Confidentiality, and Security

#### Feasibility and Impact Data

Study team members will be provided with tracking sheets to collect data on the potential participants and enrollees. For example, they will track information on who was contacted via Twitter, when a Twitter user was contacted, their Web-based engagement with the outreach message, if targeted Twitter users used the contact form on the clinical trial webpage to contact the study team, and the results of the prescreening phone call. The USC Norris team members will use tracking sheets to track information about the potential participants who were screened, their eligibility, and enrollment.

Study data will be collected using the system REDCap (Research Electronic Data Capture) at USC. REDCap is a secure Web-based application designed to support data capture for research studies [79], providing (1) an intuitive interface for validated data entry, (2) audit trails for tracking data manipulation and export procedures, (3) automated export procedures for seamless data downloads to common statistical packages, and (4) procedures for importing data from external sources. Provision of data to the IRB, National Institutes of Health (NIH), and Food and Drug Administration is facilitated by this database system. Additionally, the prescreened participants that we triage to the USC Norris team for further screening will be documented in the USC Clinical Trials Management System (CTMS) to be able to track their enrollment in one or more clinical trials.

#### Interview Data

Verbatim transcription of audio-recorded interviews with the study team members of the enrolled clinical trials will be reviewed for completeness. Transcripts of interviews will be entered, managed, and coded using Atlas.ti (ATLAS.ti Scientific Software Development GmbH), a qualitative data management computer program.

#### Data Confidentiality and Security

The data we collect will only be viewed by the study team for this project. Identifiers such as name, Twitter username, age, and gender data are collected and stored in a secure, Health Insurance Portability and Accountability Act–compliant database REDCap at USC for no longer than 1 year and will be deleted after that time. Additionally, the prescreened participants that we triage to the USC Norris team for further screening will be documented in the secure USC CTMS, which is based on the Web-based software system OnCore and designed to streamline the process of managing clinical trials. We will not store the internet protocol addresses of respondents. Names of noneligible individuals will not be maintained. The data for analysis will be deidentified.

### Data Analysis

#### Analysis of Qualitative Interview Data

To facilitate the qualitative data analysis of the interviews with the study team members of the onboarded clinical trials, we will develop an initial code list based on the interview guide. The code list will be modified throughout the coding process. Each coded transcript will be discussed line by line until the coding team (including the co-PI) comes to an agreement about code definitions and how they should be applied. Important themes will be summarized and used to understand acceptance with the social media–based intervention for cancer clinical trials. Count outcomes will be presented as median and interquartile range; nominal outcomes will be presented as N (%). We will explore participant and study team characteristics between the 2 cohorts to examine where differences might lie by including them as potential covariates in the models. Comparisons of the before- and after-time periods will be made using generalized estimating equations for appropriate outcome type (Poisson, means, and prevalence) accounting for the type of cancer.

#### Analysis of Quantitative Data

The impact of the social media intervention will be determined comparing monthly enrollment rates during the active intervention period (ie, number of people enrolled per month divided by number of people targeted on Twitter per trial disease group per month) versus the prior 10 months as historical control for each disease trial group (ie, breast cancer, colon cancer, kidney cancer, lymphoma, non-small cell lung cancer, and prostate cancer) using generalized estimating equations, accounting for intradisease random effects and trends. Analyses will be performed in SPSS v24 [40]. As this is a pilot study, *P* values are of limited use to determine group differences, so we will focus on observed effect sizes (Cohen *d*, relative risk). Additional quantitative data will be calculated using proportions of targeted Twitter users who engaged with outreach messages (measured through Twitter replies, mentions, *likes*, *retweets*, direct messages, following, and contact form use on the trial webpage). To aid in the design of larger and more definitive studies, we also intend to estimate the effect size of the number of people enrolled associated with the use of targeted social media monitoring on Twitter as a tool for enhancing cancer trial recruitment.

### Risk Analysis

#### Anticipated Risk

This research project presents minimal-risk research. We will use public user data from the social network Twitter. Identifiable information such as human subjects’ names and Twitter handles will not be included in the analysis dataset. Patient identifiers do not apply. We have implemented the following measures to ensure data and information confidentiality and to minimize risk (see Data Confidentiality and Security section). We will further abide by USC IRB regulations and the USC Privacy of Personal Information policy. In general, all data will be entered into a computer and database that is password protected. The data will be stored using appropriate, secure computer software and encrypted computers. The IRB-approved study protocol details further information on procedures for monitoring and assessing study-related concerns (Multimedia Appendix 1, page 16).

#### Anticipated Challenges

We identified a number of scientific, ethical, and regulatory challenges to this study. Refer to the IRB-approved study protocol for further information on perceived ethical and regulatory issues and how we will manage these challenges and related risk (Multimedia Appendix 1, pages 17-18).

### Dissemination of Study Findings

The study authors plan to publish the study findings in a peer-reviewed journal and at topic-related conferences (to be determined at a later date). All listed authors and/or contributors are compliant with guidelines outlined by the International Committee of Medical Journal Editors for author inclusion in a published work. Public access to the study protocol and other necessary aspects will be made available through our ClinicalTrials.gov identifier (NCT03408561). Furthermore, to support research transparency and reproducibility, we will share the deidentified research data after publication of the study results. We will share the deidentified data on Figshare, a repository where users can make all of their research outputs available in a citable, shareable, and discoverable manner. We will use a data-sharing agreement that provides for (1) a commitment to using the data only for research purposes and not to identify any individual participant and (2) a commitment to securing the data using appropriate computer software.

## Results

This study has been funded by the NCATS through a Clinical and Translational Science Award (CTSA) award (Multimedia Appendix 7). Study approval was obtained from the CIC at USC Norris (Protocol 0S-17-7; Multimedia Appendix 2) and the IRB at USC (Protocol HS-17-00811; Multimedia Appendix 3). This study is also registered at ClinicalTrials.gov (NCT03408561). Study recruitment via Twitter started in February 2018. Data collection will be completed in November 2018.

## Discussion

### Limitations

We recognize that this pilot study will not have sufficient resources to recruit a truly representative sample. Thus, the generalizability of the study results is somewhat limited. Twitter messages from locations outside of Los Angeles County, as well as messages in other non-English languages such as Spanish, and therefore nonspeakers of English, will not be included. Moreover, the social media intervention favors those with internet access and could therefore introduce potential bias into the clinical trials. Regardless of the fact that social media users “have grown more representative of the broader population” [18], Twitter users tend to be younger (40% are aged 18-29 years), college graduates, and located in urban areas [18,19], compared with the “average” study participant. It is also worth mentioning that based on Pew Research data from 2018, the percentage of Twitter users among the black population (26%) is now higher than the percentage of white (24%) and Hispanic (20%) Twitter users [18]. Recruiting via Twitter has the potential to select for this segment of the population and could therefore introduce bias. Future research will need to determine the extension of the findings and conclusions to the population at large. However, we will keep a detailed account of the environment surrounding this research and include a rich description in our final report to ensure that the study findings and the described method for implementing a centralized social media intervention at a comprehensive cancer center are transferable to other academic settings. Additionally, much of the Twitter data we use will be prospective. However, we also include retrospective social media data (ie, relevant Twitter messages sent by users in Los Angeles County 6 months before study onset). The fact that these messages are older than the messages in the prospective dataset may affect the likelihood of a targeted Twitter user engaging with the outreach message. Furthermore, the possibility remains that factors such as disease awareness months and trending news will affect the attention to outreach messages. Finally, we must consider that successful social media engagement may not necessarily correlate with clinical trial enrollment due to variables that affect the clinical trial consent and enrollment process and are unrelated to social media monitoring and outreach.

### Practical Significance

This pilot project will provide preliminary data and practical insight into the application of publicly available Twitter data to identify and recruit clinical trial participants at a comprehensive cancer center across 6 cancer disease types. If successful, the findings of this study will inform a multisite RCT to determine the efficacy of the social media intervention described here across different locations and populations. In addition, data from Twitter users and study team members of the onboarded clinical trials will be translated into a preliminary set of testable questions to further examine challenges around the use of social media monitoring in clinical trial recruitment in general and at comprehensive cancer centers.

## Appendix

Multimedia Appendix 1 Institutional review board–approved study protocol.

Multimedia Appendix 2 Approval notice provided by the clinical investigations committee at the USC Norris Comprehensive Cancer Center.

Multimedia Appendix 3 Approval notice provided by the institutional review board at the University of Southern California.

Multimedia Appendix 4 Boolean and Regex location code categories for identifying Twitter users in LA County.

Multimedia Appendix 5 Keywords and hashtags for monitoring Twitter user conversations in LA County and for identifying potential clinical trial participants.

Multimedia Appendix 6 Consent and prescreening questionnaire for targeted Twitter users who contact the study team.

Multimedia Appendix 7 National Institutes of Health summary statement of approved study. Relevant pages of peer-review report for center grant have been extracted from overall summary statement.

## Abbreviations

CIC: clinical investigations committee
CISO: Clinical Investigation Support Office
COI: conflict of interest
CTMS: Clinical Trials Management System
IRB: institutional review board
NCATS: National Center for Advancing Translational Sciences
NIH: National Institutes of Health
PHS: Public Health Service
PI: principal investigator
RCT: randomized controlled trial
REDCap: Research Electronic Data Capture
USC: University of Southern California
USC Norris: USC Norris Comprehensive Cancer Center
