# Supplementary material for: Monitoring Twitter Conversations for Targeted Recruitment in Cancer Trials in Los Angeles County: Protocol for a Mixed-Methods Pilot Study
Source: JMIR Res Protoc. 2018 Sep 25;7(9):e177. doi: 10.2196/resprot.9762 (PMC6231794; doi:10.2196/resprot.9762)
Supplement: Multimedia Appendix 1 [file resprot_v7i9e177_app1.pdf]

Research Study Protocol

The use of social media listening for targeted recruitment of Twitter users in LA County in cancer trials compared to historic recruitment data: A mixed-methods study

Funded by: National Center for Advancing Translational Science (NCATS)

A collaboration between the Southern California Clinical and Translational Science Institute (SC CTSI) and the USC Norris Comprehensive Cancer Center (USC Norris)

SC CTSI
PI: Thomas A. Buchanan, MD
Co-PI (contact PI): Katja Reuter, PhD (katja.reuter@usc.edu)
Research assistant: Namquyen Le

USC Norris
Co-Investigators:
Anthony El-Khoueiry, MD; Sarah Cole (colon cancer)
David I. Quinn, MD (prostate and kidney cancers)
Irene Kang, MD (breast cancer)
Jorge Nieva, MD (lung cancer)
Kevin Kelly, MD (lymphoma)

Biostatistics consultant: Christianne Joy Lane, PhD
Qualitative research consultant: Ricky Bluthenthal, PhD
Private sector partner: Symplur (Audun Utengen and Thomas Lee)

Study site
USC Norris Comprehensive Cancer Center
Keck School of Medicine of USC
University of Southern California (USC)
2250 Alcazar Street, CSC 200
Los Angeles, CA 90033

TABLE OF CONTENTS

1. PURPOSE OF THE STUDY 6

1.1 NAME OF INTERVENTION 6
1.2 INTENDED USE OF THE INTERVENTION 6
1.3 STUDY OBJECTIVE 6
1.4 ANTICIPATED DURATION OF THE CLINICAL INVESTIGATION 6

2. BACKGROUND 7

2.1 PROBLEM THE STUDY ADDRESSES 7
2.2 RATIONALE 7

3. STUDY PROTOCOL 9

3.1 PROTOCOL TITLE 9
3.1 PROTOCOL VERSION NUMBER AND DATE 9
3.1 PROTOCOL DESIGN 9
3.3.1 GENERAL STUDY DESIGN 9
3.3.2 STUDY FLOW DIAGRAM 10
3.3.3 ELIGIBILITY: TRIAL AND PARTICIPANT SELECTION 10
3.3.4 STUDY INTERVENTION 12
3.3.5 DATA COLLECTION 14
3.3.6 DATA SECURITY AND CONFIDENTIALITY 14
3.3.7 DATA ANALYSIS 14
3.3.8 PROCEDURES FOR MONITORING AND ASSESSING CONCERN 16

4. BENEFITS 16

5. RISK ANALYSIS 16

5.1 ANTICIPATED RISKS 16
5.2 ANTICIPATED CHALLENGES 16

6. INFORMED CONSENT 19

7. CONFLICT OF INTEREST 19

8. REFERENCES 19

APPENDIX 1: FULL TWITTER USER DATA ANALYSIS BY CANCER DISEASE TYPE IN CALIFORNIA 23

APPENDIX 2: ACCRUALS AND ACCRUAL TARGETS BY TUMOR TYPE LUNG, COLON, BREAST, PROSTATE, KIDNEY AND LYMPHOMA 24

APPENDIX 3: OVERVIEW OF TWITTER OUTREACH MESSAGE TEMPLATES 25

APPENDIX 4: STUDY WEB PAGE EXAMPLE 28

APPENDIX 5. DATA DICTIONARY 29

APPENDIX 6. QUESTIONNAIRE FOR PROSPECTIVE STUDY PARTICIPANTS 32

LIST OF ABBREVIATIONS

Co-I	Co-Investigator
HIPAA	Health Insurance Portability and Accountability Act of 1996
I	Investigator
IRB	Institutional Review Board
SM	Social media
SML	Social media listening (i.e., surveillance, monitoring)
PI	Principal investigator

PROTOCOL SYNOPSIS

Title	The use of social media listening for targeted recruitment of Twitter users in LA County in cancer trials compared to historic recruitment data: A mixed-methods study
Sponsor	N/A
Funding organization	National Center for Advancing Translational Science (NCATS)
Number of sites	1 USC Norris Comprehensive Cancer Center (USC Norris)
Rationale	Participant recruitment into clinical trials represents a major barrier to clinical and translational research and is often associated with implementation delays and high costs. This hinders the translation of scientific discoveries into interventions that improve the health of individuals and the public across populations. Several groups have demonstrated that social media (SM) such as Twitter and Facebook can be used to recruit and enroll participants efficiently into clinical studies. This study will examine the feasibility and gain preliminary data on the impact of targeted social media listening (SML) as a tool for enhancing recruitment to cancer trials (non-small cell lung cancer, colon cancer, breast cancer, prostate cancer, kidney cancer, lymphoma) among Twitter users in LA County. Targeted SML employs community-generated data from Twitter to identify prospective participants based on health interests and disease topics they mention in their Twitter messages. Those individuals are then targeted for recruitment using a combination of SM and traditional methods. Success will be compared to historical success achieved without the use of SML.
Study design	This is a mixed-methods study.
Objectives	1. Examine the feasibility (acceptance among USC Norris study team members and Twitter users in LA County) of targeted social media listening (SML) via Twitter as a tool for enhancing recruitment to cancer trials.<br>2. Gain preliminary data on the impact (i.e., numbers recruited versus projected accrual per trial disease area compared to historic recruitment) of targeted social media listening (SML) as a tool for enhancing recruitment to cancer trials among Twitter users in LA County. We will further estimate the effect size of the number of people enrolled per trial disease area associated with the use of targeted social media listening (SML) via Twitter.
Number of trials/research participants	1. 84 clinical cancer trials (all interventional trials open for recruitment to accrual for treatment of patients with non-small cell lung cancer, prostate cancer, breast cancer, colon cancer, kidney cancer, or lymphoma-- based on USC Norris data from 1/1/2017 to 07/07/2017). We may include another tumor type if the number of trials is insufficient or other tumor types and Twitter user activity emerge that are of interest to the research question.)<br>2. 160 participants (2 members per enrolled trial, PIs, study coordinators, recruitments specialists)
Selection criteria	Inclusion criteria: Eligible clinical trials must focus on one or more of the pre-selected cancer disease types, i.e., non-small cell lung cancer, prostate cancer, breast cancer, colon cancer, kidney cancer, or lymphoma (trial selection is independent of stage of disease); be a Phase 1 trial in expansion, Phase 2, or 3; be interventional trials; recruit in English; be IRB-approved and open to accrual at USC Norris; recruit for at least 9 months at the point of enrollment; and set monthly accrual target ≥1/annual accrual target ≥12.<br>Exclusion criteria: Phase 1 trials in dose escalation.
Investigational methods/Intended use	1. We will use Symplur Signals, a healthcare SM analytics platform that maintains a database of curated disease- and health-related Twitter conversations and user data, updated daily and easily sortable by SM user type (patient, physician, health care organization), location and time zone, language, disease/health interests, message content, sentiment, and SM influence. We will use the data to identify users on Twitter in LA County who mention specific disease/health-related keywords and contact them to recruit them into clinical trials. Those individuals are then targeted for recruitment using a combination of SM and traditional methods.<br>2. We will use semi-structured interviews to assess pre-and post-perspectives and concerns among PIs and research team members regarding the use SML and SM recruitment to recruit cancer trial participants.<br>3. We will use brief survey interviews to assess participant satisfaction with recruiting

	method.
Control group	Success will be compared to historical success achieved without the use of SML.
Duration of subject participation	After prospective participants use the contact form on the study webpage to contact the study team, the study team (SC CTSI) will triage the request to the disease team member at USC Norris. The team member will then contact (via phone or email) the prospective study participant for further screening.
Duration of trial participation and duration of study	Duration of trial participation: 9 months (Disease categories will be rolled consecutively, one every two weeks in the first quarter. Order will be randomized.)<br>Duration of study: 14 months (this includes data analysis and dissemination of results, i.e., submission of manuscript)
Evaluations	
Primary outcomes	1. Feasibility: Acceptance of and satisfaction with SM recruitment method among USC Norris study team members and Twitter users in LA County, number of cancer trials eligible to be enrolled in study, number of cancer disease types and studies that can be monitored for on Twitter simultaneously by the SC CTSI study team, time and effort it takes the SC CTSI and USC Norris team members to respond to the resulting inquiries and decide whether or not to bring the patient in for screening, eligibility rate of prospective participants, demographic and ethnic diversity rate of those who consented/enrolled, social media message click rate, website contact form usage rate.<br>2. Impact: Monthly observed recruitment rate vs. monthly projected accrual target by trial disease group compared to historic data. Effect size estimate of the number of people to enroll cancer trials as a result of targeted social media listening and recruitment (SML) via Twitter.
Safety evaluations	N/A
Analysis plan	1. Feasibility will be assessed by describing the acceptance of and satisfaction with SM recruitment method among USC Norris study team members and Twitter users in LA County and by describing the proportion of eligible cancer trials to be enrolled in the study, cancer disease types and studies that can be monitored for on Twitter at a time, time and effort it takes the SC CTSI and USC Norris team members to respond to the resulting inquiries and decide whether or not to bring the patient in for screening, eligible participants that can be enrolled, demographic and ethnic diversity of participants, and staffing required to monitor eligible studies.<br>2. Impact: The efficacy aim will be determined comparing monthly actual vs. projected rate of recruitment for the trial disease groups (i.e., non-small cell lung cancer, colon cancer, breast cancer, prostate cancer, kidney cancer, lymphoma) using generalized estimating equations, accounting for intra-disease random effects and trends across the 12 months (9 months exposure per trial). These results will then be put into context of the historic recruitment for these diseases at the center. We will also explore patient characteristics between the two groups to examine where differences might lie. Exploratory variables will also include the number of open trials and amendments to the trials and changes to status. Effect size estimate of the number of people to enroll cancer trials as a result of targeted social media listening and recruitment (SML) via Twitter.

1. PURPOSE OF THE STUDY

1.1 Name of intervention

This study will examine the feasibility and gain preliminary data on the impact of targeted social media listening (SML) as a tool for enhancing recruitment to cancer trials (non-small cell lung cancer, colon cancer, breast cancer, prostate cancer, kidney cancer, lymphoma) among Twitter users in LA County.

1.2 Intended use of the intervention

We will use the Symplur Signals database to identify in real time people in California who mention one or more of the pre-selected cancer topics (non-small cell lung cancer, colon cancer, breast cancer, prostate cancer, kidney cancer, lymphoma) on Twitter. Symplur Signals is a healthcare SM analytics platform that maintains a database of curated disease- and health-related Twitter conversations and user data, updated daily and easily sortable by SM user type (patient, physician, health care organization), location and time zone, language, disease/health interests, message content, sentiment, and SM influence. We will use the data to identify users on Twitter in LA County who mention specific disease/health-related keywords and contact them to recruit them into clinical trials. Those individuals are then targeted for recruitment using a combination of SM and traditional methods.

1.3 Study objectives

1.3.1 Examine the feasibility (acceptance among USC Norris study team members and Twitter users in LA County) of targeted social media listening (SML) via Twitter as a tool for enhancing recruitment to cancer trials.

1.3.2 Gain preliminary data on the impact (i.e., numbers recruited versus projected accrual compared to historic recruitment) of targeted social media listening (SML) as a tool for enhancing recruitment to cancer trials among Twitter users in LA County. Estimate the effect size of the number of people enrolled associated with the use of targeted social media listening (SML) via Twitter as a tool for enhancing recruitment to cancer trials.

1.4 Anticipated duration of the clinical investigation

We estimate 14 months to complete the proposed study including the data analysis and dissemination. The intervention exposure time is 9 months per enrolled trial. Disease categories will be rolled consecutively, one every two weeks in the first quarter. Order will be randomized.

2. BACKGROUND

2.1 Problem the study addresses

In 2012, the Institute of Medicine recognized the seriousness of the clinical trial participation problem [1] and released a report that identified numerous barriers, including the lack of awareness among patients that clinical trials are available. Identifying and recruiting the full complement of participants into a trial is challenging and costly. Nearly a third of the time and resources dedicated to clinical trials is spent on study participant recruitment [2]. Despite this substantial investment, many trials fail to meet enrollment targets. Authors of randomized trials have reported that nearly 60% of them had either failed to meet their recruitment target or required an extended recruitment period to do so [3-8]. Therefore, we propose to provide evidence for the feasibility of a new clinical trial recruitment solutions to identify and recruit participants into cancer clinical trials at USC Norris.

2.2 Rationale

Studies suggest that social media (SM) such as Twitter and Facebook serve as a useful tool to recruit research participants in a variety of disease and health contexts. SM are widely accessible Web-based and mobile technologies that allow users to view, create and share information online and to participate in social networking [9]. The SM platform Twitter, for example, was successfully used to recruit cancer patients [10], young women into nutrition research studies [11], and adolescents with type 1 diabetes and their parents [12]. A recent scoping review concluded that SM can be more efficient than traditional recruitment methods for hard-to-reach populations and observational studies [13]. Paid advertisement (ads) on Facebook have shown to lead to higher enrolment rates than messages on Twitter, where ads about clinical trials and recruitment are not permitted [13]. However, in previous studies, SM were used as unidirectional communication tools to distribute messages from academic institutions to communities that did not take into account community-generated user data from SM to identify prospective study participants who have expressed interest in specific disease/health topics and to inform and guide recruitment strategies. Many research institutions use SM to distribute information about biomedical research and ongoing research activities to the public but most messages are purely informational and designed to increase transparency about activities. We propose to develop and evaluate a complementary SM monitoring approach that allows research institutions to utilize community-generated SM data from Twitter in order to identify prospective participants based on health interests and disease topics they mention on Twitter and to recruit them into clinical trials.

SM monitoring describes the process of gaining insights from SM community-generated data such as users' messages, profile descriptions, and locations. The proposed SM intervention is based on the dissonance-based health behavior model [14] and hypocrisy paradigm [15,16] that was found to be the most commonly applied research paradigm and was most effective at inciting change across a range of health behaviors [17]. After people express public support for something (e.g., a disease type, clinical trial), they become motivated to act in a way that is consistent with their professed beliefs. If a person says positive things about a clinical trial and then declines to participate in a clinical trial, that creates cognitive dissonance, which may be uncomfortable. To reduce the dissonance, people who have said positive things about clinical trials may be more likely to enroll in a clinical trial (or at least express some interest in enrolling).

Previous studies have examined the use of SM monitoring data via Twitter and other SM in different contexts such as public health surveillance [e.g., 18-20]; public health safety and

emergency response operations [e.g., 21,22]; tobacco regulatory sciences [e.g., 23-25]; and monitoring pharmaceutical products, potential drug interactions and adverse events [e.g., 26-29]. The feasibility of using SM monitoring in clinical research is supported by limited reports from the pharmaceutical industry [30]. However, sponsors have reported that the lack of experienced vendors, Clinical Research Offices and internal teams to conduct SM monitoring to inform clinical research is the main barrier to the adoption of SM monitoring [30].

Focus on Twitter and cancer trials: We propose to focus on the SM platform Twitter and cancer clinical trials for the following reasons: Previous studies provide evidence of “a rapidly evolving network of cancer patients engaged in information exchange via Twitter” [31, 32] that can serve as a powerful and important tool in implementing and disseminating critical messages to the community in real-time” [31]. The increasing use of Twitter among members of the cancer disease community is evidenced by the growing use of specific hashtags [32-37]. A hashtag is a user-generated word or phrase preceded by a hash or pound sign (#) and used to identify messages on a specific topic on Twitter and other SM platforms. For example, rather than #breastcancer or #bcancer, the most widely recognized hashtag for breast cancer is currently #BCSM (Breast Cancer Social Media). Furthermore, Twitter is a public social network which makes it an ideal candidate to test the proposed research idea. By default, basic Twitter account information such as the profile name, description and location are public unless a user decides to opt out and make his/her account private. SM such as Twitter are also a “rich and promising avenue for exploring how patients conceptualize and communicate about their specific health issues” [38]. A study on lung cancer-related Twitter messages, for example, found that these messages focused on support, prevention, and clinical trials, and were predominantly authored by individuals [39]. Therefore, we hypothesize that the Twitter network can be used for precision recruitment to identify, engage and recruit prospective study participants, and that previously reported recruitment rates via Twitter can be increased through SM monitoring.

Research questions we aim to answer with this study:

I. **How feasible is the application of SML to enhance recruitment for clinical trials among Twitter users in LA County? This will be measured as follows**:
   a. How many PI’s are willing to participate in this study?
   b. How many cancer trials that meet the eligibility criteria can be enrolled?
   c. What are reasons for not enrolling eligible trials?
   d. How many trials and disease categories can be monitored at a time? Monitoring means that the SC CTSI study team listen to conversations on Twitter that mention the select disease terms (i.e., non-small cell lung cancer, colon cancer, breast cancer, prostate cancer, kidney cancer, lymphoma) to identify potential study participants in LA County.
   e. How does the intervention affect the enrolled study and study staff?
   f. How much time and efforts does it take to respond to the resulting inquiries and decide whether or not to bring the patient in for screening?
   g. How does the intervention affect prospective participants’ satisfaction?
   h. How many prospective participants are eligible?
   i. How diverse are enrolled participants? (demographic and ethnic diversity)

II. **How effective is the application of SML to enhance recruitment for clinical trials among Twitter users in LA County?**
   a. How does the enrollment rate that results from SML on Twitter compare to historic recruitment data? Measured actual versus projected accrual per month at USC Norris.

3. STUDY PROTOCOL

3.1 Protocol title

The use of social media listening for targeted recruitment of Twitter users in LA County in cancer trials compared to historic recruitment data: A mixed-methods study.

3.2 Protocol version number and date

Version 5, Jan 23, 2018.

3.3 Study design

3.3.1 General study design

We will use a mixed-methods interrupted time series study design with a before and after intervention at the USC Norris Comprehensive Cancer Center (USC Norris). This study will examine the feasibility and gain preliminary data on the impact of targeted social media listening (SML) as a tool for enhancing recruitment to cancer trials (non-small cell lung cancer, colon cancer, breast cancer, prostate cancer, kidney cancer, lymphoma) among Twitter users in LA County. Disease categories will be randomized and rolled in consecutively, one every two weeks in the first quarter. The order will be randomized.

We aim to recruit all interventional trials in the select disease areas (84; based on USC Norris data from 1/1/2017 to 07/07/2017) open for recruitment to accrual for treatment of patients with non-small cell lung cancer, prostate cancer, breast cancer, colon cancer, kidney cancer, or lymphoma) at USC Norris. We may include another tumor type if the number of trials is insufficient or other tumor types and Twitter user activity emerge that are of interest to the research question. Disease categories will be randomized and rolled in consecutively, one every two weeks in the first quarter. Once a study is enrolled it stays on for the period of this study. Each trial will be exposed to the intervention for 9 months. This design was chosen to fit within the triage protocol of USC Norris and cause minimal disruption to the staff. Success will be compared to historical success achieved without the use of SML.

Outcomes will be the feasibility of the study described by the acceptance of and satisfaction with SM recruitment method among USC Norris study team members and Twitter users in LA County and by describing the proportion of eligible cancer trials to be enrolled in the study, cancer disease types and studies that can be monitored at a time, eligible participants that can be enrolled, diversity of participants, staffing required to monitor eligible studies, burden of recruitment/cost of intervention.

- <u>Post-qualitative interviews with study team members</u> will be undertaken to explore trial participants' views and experiences of the trial methods and intervention (principal investigators, clinical research coordinators, recruitment specialists). The interview guide is under development and will be submitted to the USC HSCIRB for review. Qualitative research expert Ricky Bluthenthal, PhD (consultant on this project) will advise this part of the study. We will gather a range of data. Please see **Table 3** that lists all outcomes in the *Data analysis* section 3.3.7.

Recruitment of USC Norris clinical trial team members: As we enroll cancer clinical trials in this study, the study co-PI (Dr. Katja Reuter) will contact the cancer trial PI via email (using USC's email system) to invite the PI and one additional study member (e.g., clinical research coordinator, recruitment specialist) to participate in an interview.

- Brief survey interviews with contacted Twitter users will be conducted by the study teams with prospective study participants who contacted the study team in response to SM recruitment. The goal of the survey is to collect demographic information about the prospective study participants and to better understand their perception of the SM recruitment method used. Qualitative research expert Ricky Bluthenthal, PhD (consultant on this project) will also advise this part of the study. (See **Appendix 6** for the full questionnaire)

Additionally, we will look at the impact of the SM intervention measured by monthly observed recruitment rate vs. monthly projected accrual target. We will calculate the effect size estimate of the number of people to enroll cancer trials as a result of targeted social media listening and recruitment (SML) via Twitter.

3.3.2 Study flow diagram

The diagram shows the intervention of social media listening (SML) and clinical trial recruitment via Twitter (see page 11).

3.3.3 Eligibility: Trial and participant selection

TRIAL

General characteristics of clinical trials to be enrolled: Eligible trials will focus on one the six pre-selected cancer disease types, i.e., non-small cell lung cancer, colon cancer, breast cancer, prostate cancer, kidney cancer, lymphoma. This trial selection is independent of stage of disease. The disease categories we selected are based on a preliminary Twitter user data analysis in California (Table 1, **Appendix 1**) and the number of prospective, recruiting trials at USC Norris (**Appendix 2**).

Eligible clinical trials must:

- focus on one of the following disease categories: non-small cell lung cancer, colon cancer, breast cancer, prostate cancer, kidney cancer, lymphoma,
- be a Phase 1 trial in expansion, Phase 2, or 3,
- be interventional trials,
- recruit in English,
- be IRB-approved and open to accrual at USC Norris,
- recruit for at least 9 months at the point of enrollment, and
- set monthly accrual target ≥1/ and annual accrual target ≥12.

Exclusion criteria: Phase 1 trials in dose escalation.

Table 1. Total social media activity on Twitter among English-speaking populations from January 1, 2016 to January 30, 2017 in California for pre-select cancer disease terms. See

Appendix 1 for detailed analysis of Twitter user activity by cancer type. *Note: The preliminary Twitter user activity data were provided by our private-sector partner Symplur for free. At the onset of this project, we will have access to the Symplur Signals database and will be able to refine the search focused on Twitter user for LA County.*

Location	California
Total number of Twitter messages (in English) that include pre-selected disease terms	159,396
Total number of Twitter users who used pre-selected cancer disease terms in one or more of their Twitter messages	36,502

<u>Anticipated number of clinical trials</u>: Based on a preliminary data analysis (**Appendix 2**), at this point in time there are 84 clinical trials open at USC Norris that meet the criteria for inclusion in this study. SML will be used to identify potential participants for all these trials.

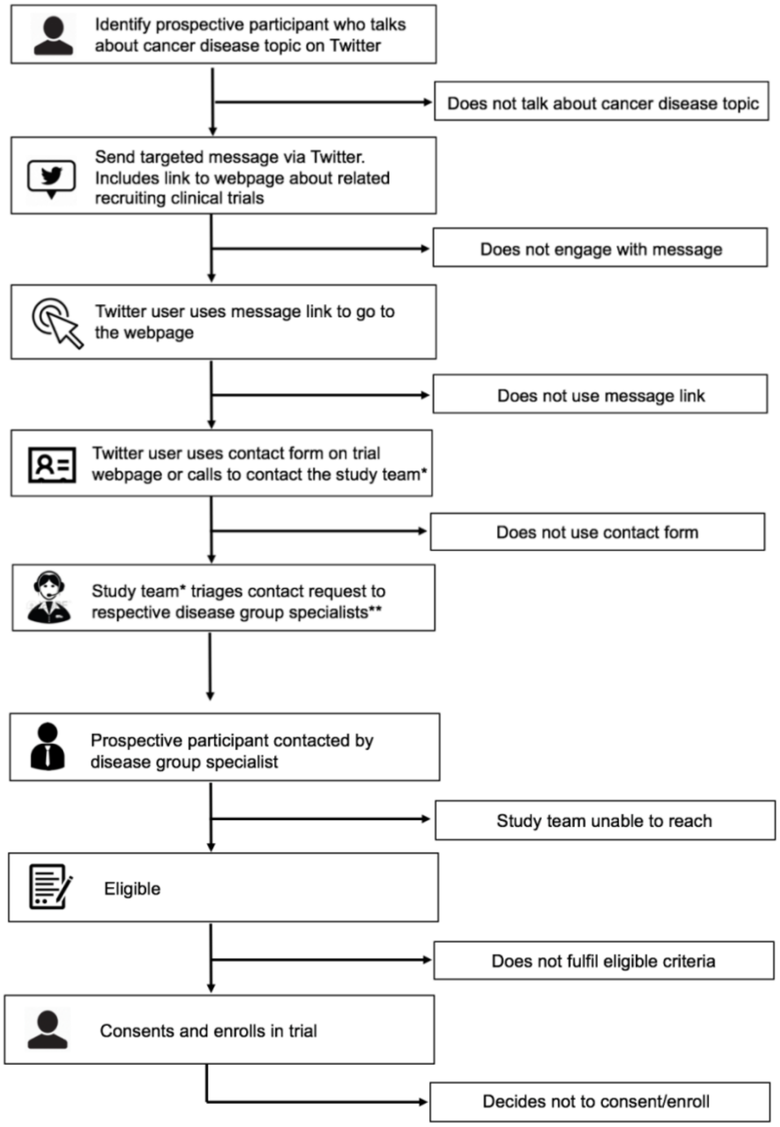

* Katia Reuter or Namquyen Le
** Sarah Cole; Anthony El-Khoueiry, MD (colon cancer); Tanya Dorff, MD (prostate and kidney cancers); Irene Kang, MD (breast cancer); Jorge Nieva, MD (lung cancer); Kevin Kelly, MD (lymphoma)

Figure 1. Study flow diagram of study design, procedures and stages for intervention A.

PARTICIPANTS

General characteristics of the proposed participant population(s): Any Twitter user who mentions one or more of the pre-selected cancer disease topics will be contacted via Twitter.

Anticipated number of research participants: We anticipate increasing the number of participants enrolled per trial and per month based on the following historic data from the past five years (see **Appendix 2**):

- Non-small cell lung cancer: Minimum of 2 participants per month
- Colon cancer: Minimum of 2 participants per month
- Prostate cancer: Minimum of 3 participants per month
- Breast cancer: Minimum of 2 participants per month
- Kidney cancer: Minimum of 1 participants per month
- Lymphoma: Minimum of 1 participants per month

Inclusion criteria for prospective trial participants: We will include all prospective trial participants in this study that come from Twitter in response of our SM recruitment interventions, provided they meet the specific trial's eligibility criteria.

Exclusion criteria for prospective trial participants: Persons who do not meet the eligibility criteria of any of the trials open to accrual will be excluded from participation, and persons who may be eligible (e.g., disease/histology, stage, prior treatment) but do not meet additional trial-specific requirements such as insurance or allergy to drug). These may vary by clinical trial. We will count these people as engaged but not enrolled and document the specific reasons.

Screening procedures**:** We will use the eligibility criteria as outlined above to verify clinical eligibility for participation in the study.

3.3.4 Study intervention

We will use Symplur Signals, a healthcare SM analytics platform that maintains a database of curated disease- and health-related Twitter conversations and user data, updated daily and easily sortable by SM user type (patient, physician, health care organization), location and time zone, language, disease/health interests, message content, sentiment, and SM influence.

We will use prospective data as well as retrospective data (last 6 months at the time of project implementation) to identify users on Twitter in LA County who mention specific cancer disease keywords and/or hashtags and contact them to recruit them into clinical trials. A hashtag is a word or phrase preceded by a hash or pound sign (#) that is used to identify messages on a specific topic on Twitter (e.g., #diabetes, #stroke). Table 2 lists a limited overview of the keywords or hashtags we will use for monitoring during the project period. The full list of keywords and hashtags relevant to the six cancer disease terms will be extracted from the Symplur database at the onset of the project because part of the information is proprietary and only accessible after purchasing data access.

Those individuals are then targeted for recruitment using a combination of SM and traditional methods. We aim to recruit all trials (84) open for recruitment to accrual for treatment of patients with non-small cell lung cancer, prostate cancer, breast cancer, colon cancer, kidney cancer, or lymphoma at USC Norris. Disease categories will be randomized and rolled in consecutively, one every two weeks in the first quarter. Each trial will be exposed to the intervention for 9 months.

Table 2. Limited list of keywords or hashtags that will be used for monitoring Twitter user conversations in LA County during the project period. (Reference: https://www.symplur.com/

Cancer disease type	Keywords and hashtags to be monitored
Breast cancer	#breastcancer<br>#BCSM (breast cancer and social media)<br>breast [AND] cancer
Colon cancer	#CCSM<br>#ColonCancer<br>#ColorectalCancer<br>colon [AND] cancer<br>colorectal [AND] cancer
Kidney cancer	#KidneyCancer<br>kidney [AND] cancer
Lymphoma	#Lymphoma
Non-small cell lung cancer	#LCSM (lung cancer and social media)<br>#LungCancer<br>lung [AND] cancer [AND] non-small cell
Prostate cancer	#PCSM (prostate cancer and social media)<br>#ProstateCancer<br>prostate [AND] cancer

We will send identified Twitter users IRB-approved messages via the Twitter account we create for the purpose of this project @USCNorrisTrials to attract their attention, engage, and recruit them into the enrolled clinical trials. Message example: @JaneDoeA, this may be of interest to you. Researchers @USC are looking for participants for this new #LungCancer trial [Link to clinical trial webpage]. See **Appendix 3** for overview of Twitter outreach message templates.

Outgoing messages will be public and organic (not advertised). Twitter does not permit paid advertisement for clinical trials. The messages will refer recipients to the respective cancer disease trial group webpage hosted by the USC Clinical Studies Directory. For example, a message may promote lung cancer trials at USC Norris and refer Twitter users to the webpage that lists all recruiting lung cancer trials at USC Norris. An easy-to-use contact form that asks for the name and email can be used by the user to contact the study team. See **Appendix 4** shows an example of such a webpage. The web page content used for this research will be submitted to the IRB once we enroll the trials and know their focus and consent information. The consent information will guide the content we use on the web pages.

Using the contact form on the web page will trigger an email to the SC CTSI study team (Dr. Reuter, Namquyen Le) who will then triage the request to the respective cancer disease contact at USC Norris. See also Figure 1. For the purpose of tracking, we will tag each message through an element descriptor in the Hypertext Markup Language. This will allow us to determine the recruitment results by individual and message.

There is no active control group in this study. Success will be compared to historical success achieved without the use of SML at USC Norris for trials in the selected disease cancer categories.

3.3.5 Data collection

The study teams at SC CTSI and USC Norris will be provided with tracking sheets to collect data on the prospective participants and enrollees. For example, the SC CTSI team will track information on who was contacted via Twitter, when a Twitter user was contacted, their online engagement with the recruitment message, and if they used the contact form on the clinical trial web page to contact the study team. The USC Norris team will use a tracking sheet to track information about the prospective participants that were screened, their response, and whether an enrollee of a USC Norris cancer trial had been contacted through the investigators' program. Please see **Table 3** for an overview of all primary outcome measures and **Appendix 5** for the data dictionary that list all metrics the study team will use to characterize the success of the intervention.

Study data will be collected using the system REDCap (Research Electronic Data Capture) at USC. REDCap is a secure, web-based application designed to support data capture for research studies [40], providing: 1) an intuitive interface for validated data entry; 2) audit trails for tracking data manipulation and export procedures; 3) automated export procedures for seamless data downloads to common statistical packages; and 4) procedures for importing data from external sources Provision of data to the IRB, NIH, and FDA is facilitated by this database system.

Verbitum transcription of audio recorded interviews will be reviewed for completeness. Transcripts of interviews will be entered and managed using Atlas.ti, a qualitative data management computer program.

3.3.6 Data security and confidentiality

The data will only be viewed by the study team for this project. Identifiers such as name, Twitter user name, age and gender data are collected and stored in a secure, HPAA-compliant database REDCap at USC for no longer than 1 year and will be deleted after that time. We won't store the IP addresses of respondents. Names of non-eligible individuals will not be maintained. The data for analysis will be de-identified.

To support research transparency and reproducibility, we will share the de-identified research data after publication of the study results. We will share the de-identified data on Figshare (Figshare.com), a repository where users can make all of their research outputs available in a citable, shareable and discoverable manner. We will use a data-sharing agreement that provides for: (1) a commitment to using the data only for research purposes and not to identify any individual participant; and (2) a commitment to securing the data using appropriate computer software.

3.3.7 Data analysis

<u>Feasibility</u> will be assessed by describing the acceptance of and satisfaction with the SM recruitment method among USC Norris study team members and Twitter users in LA County, number of cancer trials eligible to be enrolled in study at the time of this study (current estimate of 84 is based on latest data from USC Norris), number of cancer disease types and studies that can be monitored for on Twitter simultaneously by the SC CTSI study team, time and effort it takes the SC CTSI and USC Norris team members to respond to the

resulting inquiries and decide whether or not to bring the patient in for screening, eligibility rate of prospective participants, demographic and ethnic diversity rate of those who consented/enrolled, social media message click rate, website contact form usage rate, social media message click rate, website contact form usage rate. **Table 3** lists all primary outcomes measures we intend to use.

To facilitate qualitative data analysis, we will develop an initial code list based on the interview guide. The code list will be modified throughout the coding process. Each coded transcript will be discussed line by line until the coding team came (Drs. Reuter and Bluthenthal) to an agreement about code definitions and how they should be applied. Important themes will be summarized and used to understand satisfaction with SM-based recruitment strategies for cancer-related clinical trials.

Count outcomes will be presented as median and interquartile range, nominal outcomes will be presented as N (%). We will explore patient and study characteristics between the two cohorts to examine where differences might lie by including them as potential covariates in the models. Comparisons of the before- and after time periods will be made using generalized estimating equations for appropriate outcome type (Poisson, means, prevalence) accounting for type of cancer.

The **efficacy** aim will be determined comparing monthly actual vs. projected rate of recruitment for the trial disease groups (i.e., non-small cell lung cancer, colon cancer, breast cancer, prostate cancer, kidney cancer, lymphoma) and compared to historic data by trial disease group using generalized estimating equations, accounting for intra-disease random effects and trends across the 12 months (9 months exposure per trial). Statistical analyses of primary aims will be carried out by Dr. Christianne Joy Lane, an SC CTSI biostatistician. Analyses will be performed in SPSS v24 [41]. As this is a pilot study, p-values are of limited use to determine group differences, so we will focus on observed effect sizes (Cohen's D, Relative Risk).

Table 3 lists all primary outcomes measures we intend to use. In addition, **Appendix 5** (**data dictionary**) provides the full set of metrics we will use to assess the success of the intervention, which includes additional success metrics such as number of contacts solicited, number of contacts who acknowledge the solicitation, number of social media contact/recruitment messages sent, day/time of contact, day/time of response.

Table 3. Primary and secondary outcomes and definitions.

Primary outcome measures	Definition and calculation
FEASIBILITY	
Feasibility	• Number of cancer clinical trials that meet study criteria and can be sampled<br>• Number of cancer disease types and studies that can be monitored for on Twitter simultaneously by the SC CTSI study team<br>• Characteristics of enrolled trials such as disease type, recruitment phase, clinical phase, target population and age<br>• Time and effort it takes the SC CTSI and USC Norris team members to respond to the resulting inquiries and decide whether or not to bring the patient in for screening<br>• Perspectives on clinically relevant increase in recruitment/Number of additional participants that studies can process
Eligibility rate of enrolled	Number of people eligible per month/Number of people screened per month (i.e.,

trial participants	who spoke with the study team)
Diversity of enrolled participants	Number of people of a specific racial/ethnic background enrolled per month/number of all people enrolled per month
Message click rate	Number of times a message link was clicked/Number of message impressions
Website contact form usage rate	Number of times a contact form was used on a trial webpage/Number of webpage sessions *(Sessions: Group of interactions a user takes on a webpage or website within a given time frame, e.g., page views, clicks.)*
User acceptance and satisfaction (%)	• Number of USC Norris study team members who are satisfied with the SM monitoring and recruitment intervention/Number of all study team members engaged<br>• Number of PIs that are willing to participate (acceptance by PIs)<br>• Number of trial participants enrolled (Twitter users in LA County) who are satisfied with the SM monitoring and recruitment intervention/Number of all people engaged (i.e., who spoke with the study team)
IMPACT AND EFFICACY	
Enrolment rate per month	• Number of people enrolled per month/Number of people contacted on Twitter per trial per month<br>• Number of people triaged and enrolled in another trial per month/Number of people contacted on Twitter per trial per month

3.3.8 Procedures for monitoring and assessing concern

Through weekly visits, the SC CTSI study team (Dr. Reuter and Namquyen Le) will be in close contact with the disease teams at USC Norris to coordinate the study. Any concerns that may be raised by either the participating cancer trial teams and disease group specialists at USC Norris or the participants that came from Twitter will be discussed with the study PIs (Drs. Thomas Buchanan and Katja Reuter), the study lead at USC Norris (Dr. Anthony El-Khoueiry) and the expert consultants on the team (as appropriate) and addressed immediately. The IRB will be involved immediately as well in case a reportable event occurs.

Additionally, Dr. Katja Reuter (co-PI and point of contact) will report any action resulting in a temporary or permanent suspension or delay of the study to the IRB and to the Office of Clinical Research. Dr. Reuter will be responsible for reporting any reasons outside the planned study design such as incompliance with the protocol or if there is any delay in the initiation of the study due to administrative reasons.

4. BENEFITS

There are no benefits to the individual participants. Generally, this study may shed light on perspectives on targeted social media recruitment methods via Twitter using surveillance and outreach techniques and estimates of effective size.

5. RISK ANALYSIS

5.1 Anticipated risks

The proposed project presents minimal-risk research. We will use public user data from the social network Twitter. Human subject’s names will not be included in the data set. Patient identifiers don’t apply. We take a number of steps to ensure data and information confidentiality

and to minimize risk. We will further abide by USC IRB regulations and the USC Privacy of Personal Information policy (https://policy.usc.edu/info-privacy/). In general, all data will be entered into a computer and database that is password protected. The data will be stored using appropriate, secure computer software and encrypted computers. Please see sections 3.3.4 and 3.3.4 on data collection, data security and confidentiality for more detail.

5.2 Anticipated challenges

We identified a number of scientific, ethical, and regulatory limitations and barriers to this study. **Table 4** lists perceived issues and how we propose to manage these challenges and related risk.

Table 4. Scientific, ethical, and regulatory limitations and barriers, and proposed solutions for this research project.

Barrier	Proposed solutions
SCIENTIFIC ISSUES	
Obtaining IRB approval for individual clinical trials to be enrolled in the study.	The USC Norris team including members from the USC Norris IRB (Susan Groshen) have been an integral part of the protocol development. Additionally, we consulted the co-director of the Clinical Investigations Support Office at USC Norris and chair of Keck's Institutional Review Board who suggested creating an "umbrella" IRB for this study that would include all trials enrolled in this study and reporting the clinical trials that will be enrolled in this study under this protocol. This document will serve as the overall ("umbrella") study protocol.
Lack of familiarity among study teams about digital tools and misconceptions about regulations	We will educate investigators and research staff about the potential and limitations of SM, e.g., there are no regulations or case laws that govern what media may be used to reach prospective study participants. Additionally, we will conduct a qualitative study as outlined in this protocol to gather information on perceptions of and acceptance of the proposed social media intervention to enhance recruitment into clinical trials.
Effect size for an improvement in clinical trial recruitment is unknown	We designed this pilot study to allow us to collect preliminary feasibility data (acceptability) and to estimate the effect size of the proposed SM recruitment intervention. We plan to use the preliminary data in support of a larger grant proposal (U01) to the National Center for Translating Science (NCATS).
Managing volume of incoming requests through clinical trial web pages	First point of contact will be the main study team (Dr. Reuter and Namquyen Le). If a Twitter user uses the contact form on a trial webpage to contact the study team, the request will trigger an email to Dr. Reuter and Namquyen Le. They will triage the requests to the relevant disease group at USC Norris. In case of high volume, we will work closely with the affected disease group team at USC Norris to assist with screening of requests and prospective participants. More specifically, we will add a pre-screening form to the respective clinical trial disease web page that will allow prospective participants to self-report key eligibility-related questions. This information will help the study team to determine eligibility and next steps. An automated algorithm developed by the SC CTSI will classify responses as concordant, discordant, or neutral for study eligibility. This approach will allow us to extend the capacity of the involved study team members by prioritizing and focusing enrollment efforts on those individuals that may be most likely to meet final eligibility criteria.
Differentiating between enrollees who have been contacted via the Twitter intervention vs. those who come in through regular channels	Our data collection approach will allow us to differentiate between enrollees who have been contacted via the Twitter intervention vs. those who come in through regular channels. First, we will collect publically available information about the Twitter users including their Twitter name, location, and Twitter account description. Second, we will correlate this information where possible with the name and email information users provide on the clinical trial web pages used in this research project. At this point, the SC CTSI team will triage the contact request to the USC Norris team that will contact the prospective trial participant to perform the eligibility screening. The USC Norris team will be

	provided with a tracking sheet to track additional information about the prospective participant including their Twitter name (if they came through Twitter). This will allow us to link up the initial contact data with the enrollee data and to differentiate between enrollees who have been contacted via the Twitter intervention vs. those who come in through regular channels. See also Appendix 5, an overview of the success metrics used for data collection.
ETHICAL AND REGULATORY ISSUES	
Bias in online recruitment. Online patient communities and SM users may exclude certain demographics, e.g., minorities, older adults.	Recent data indicate that digital and SM reach diverse groups and demographics. The use of the Internet as a top source for clinical research information has increased significantly (46% in 2013) while the use of mass media has declined (newspaper, radio, TV; 39% in 2013) [42,43]. More than 40% of the public reports that they have used SM to learn about clinical research, with Facebook topping the list [44]. Half of people over 50 and more than a third of people over 65 frequent social networking sites such as Twitter [45]. SM is used equally by 65% of whites, 65% of Hispanics and 56% of African-Americans [46,47,48]. SM is also used across socioeconomic classes as more than half (56%) of those living in the lowest-income households use SM [49]. However, to generate more insight, we will track the diversity of study participants and adjust our strategy if necessary.
Non-invasive, non-coercive, and compliant design of recruitment materials	Our recruitment materials including the SM messages on Twitter will comply with existing IRB and applicable FDA regulations related to human subject research. This means adherence to 21 CFR §56 as explained in the FDA's longstanding document titled, "Recruiting Study Subjects – Information Sheet, Guidance for Institutional Review Boards and Clinical Investigators".
Handling private health information (PHI) by SM users in response to our clinical trial messages	The following response message example demonstrate our response strategy: "Dear [@mention]: To protect your privacy, we suggest you delete your message. Please contact us directly URL"
Handling data privacy and security concerns	According to the Twitter privacy statement, information shared on Twitter is public information (e.g., Twitter name, Twitter messages, location) unless a user opts out and makes his/her account private. However, we are aware that just because Twitter content is publicly available it does not mean that the producers of such content intended it to be utilized by anyone, and probably rarely think of researchers as a target audience.<br><br>Ethical guidelines recommend a proportionate approach to ethics assessment, which advocates for risk mitigation strategies that are proportional to the magnitude and probability of risks. We will use privacy notices and disclaimers as suggested by Bender et al. as we reach out to prospective study participants. Example of notice: "The security of social media is not guaranteed. Contact us about the study. Don't post if concerned about privacy." [53]. Bender et al. draw on the principles of Privacy by Design (PbD), a globally recognized standard for privacy protection.<br><br>Additionally, a recent paper by authors from the University of Harvard suggests using nonexceptionalist methodology for assessing social media recruitment [54]. They suggest striving to "normalize social media recruitment techniques while remaining sensitive to their potentially novel aspects by, first, making their resemblance to more traditional offline recruitment explicit; second, applying the appropriate ethical considerations and scrutiny; and third, determining whether social media recruitment differs from offline recruitment in ways that warrant further review."

6. INFORMED CONSENT

Informed consent will be obtained in accordance with the Declaration of Helsinki, ICH GCP, US code of federal regulations for protection of human subjects (21 cfr 50.25[a,b], cfr 50.27, and cfr part 56, subpart a), the health insurance portability and accountability act (HIPAA), and local regulations at USC and USC Norris.

See **Appendix 6** for the informed consent form we propose to use for the prospective clinical trial participants who we engage in response to the social media intervention via Twitter. We will apply for a waiver of written consent. A properly executed, oral, informed consent will be obtained from each Twitter user who prior to entering his/her data into this trial. Twitter users will be interviewed by one of the USC Norris disease experts. Information will be given in both oral and written form (via email if provided) and participants will be given ample opportunity to inquire about details of the study.

7. CONFLICT OF INTEREST

Audun Utengen and Thomas Lee from the private-sector partner Symplur will serve as consultants on the project. We have involved the USC Office of Compliance to manage the conflict of interest (COI) from our private-sector partners in close. USC complies with the PHS regulations on Responsibility of Applicants for Promoting Objectivity in Research for which PHS Funding is Sought (42 C.F.R. Part 50, Subpart F). All disclosed conflicts will be reviewed by USC's COI Review Committee (CIRC) and either eliminated or managed prior to commencing research. Audun Utengen and Thomas Lee will not be involved in the data collection, analysis and interpretation. The study PI's, co-investigators and expert consultants don't report any COI at this point in time. Additionally, the project team will fully disclose any conflicts in presentations and publications.

8. REFERENCES

1. Public Engagement and Clinical Trials: New Models and Disruptive Technologies: Workshop Summary. Washington, DC: National Academies Press (US); 2012.
2. Web-Based Patient Recruitment: Best Opportunity to Accelerate Clinical Trials, Cutting Edge Information, Durham, NC.
3. Watson JM, Torgerson DJ (2006) Increasing recruitment to randomised trials: a review of randomised controlled trials. BMC Med Res Methodol 6: 34.
4. Langford A, Resnicow K, An L. Clinical trial awareness among racial/ethnic minorities in HINTS 2007: sociodemographic, attitudinal, and knowledge correlates. Journal of health communication. 2010;15(Suppl 3):92–101.
5. Brown M, Moyer A. Predictors of awareness of clinical trials and feelings about the use of medical information for research in a nationally representative US sample. Ethnicity & health. 2010;15:223–236.
6. Heller C, Balls-Berry JE, Nery JD, et al. Strategies addressing barriers to clinical trial enrollment of underrepresented populations: A systematic review. Contemp Clin Trials. 2014;39:169–182.
7. Shavers-Hornaday VL, Lynch CF, Burmeister LF, Torner JC. Why are African Americans under-represented in medical research studies? Impediments to participation. Ethnicity & health. 1997;2:31–45.

8. Leiter A, Diefenbach MA, Doucette J, Oh WK, Galsky MD. Clinical trial awareness: Changes over time and sociodemographic disparities. Clin Trials. 2015 Jun;12(3):215-23. doi: 10.1177/1740774515571917.
9. Dizon DS1, Graham D, Thompson MA, Johnson LJ, Johnston C, Fisch MJ, Miller R. Practical guidance: the use of social media in oncology practice. J Oncol Pract. 2012 Sep;8(5):e114-24. doi: 10.1200/JOP.2012.000610.
10. Sygna K, Johansen S, Ruland CM. Recruitment challenges in clinical research including cancer patients and their caregivers. A randomized controlled trial study and lessons learned. Trials. 2015 Sep 25;16:428. doi: 10.1186/s13063-015-0948-y.
11. Leonard A, Hutchesson M, Patterson A, Chalmers K, Collins C. Recruitment and retention of young women into nutrition research studies: practical considerations. Trials. 2014 Jan 16;15:23. Doi: 10.1186/1745-6215-15-23.
12. Hagger V, Trawley S, Hendrieckx C, Browne JL, Cameron F, Pouwer F, Skinner T, Speight J. Diabetes MILES Youth-Australia: methods and sample characteristics of a national survey of the psychological aspects of living with type 1 diabetes in Australian youth and their parents. BMC Psychol. 2016 Aug 12;4(1):42. doi: 10.1186/s40359-016-0149-9.
13. Topolovec-Vranic J, Natarajan K. The Use of Social Media in Recruitment for Medical Research Studies: A Scoping Review. J Med Internet Res 2016;18(11):e286. DOI: 10.2196/jmir.5698.
14. Stice E, Shaw H, Becker CB, Rohde P. Dissonance-based Interventions for the Prevention of Eating Disorders: Using Persuasion Principles to Promote Health. Prevention science: the official journal of the Society for Prevention Research. 2008;9(2):114-128. doi:10.1007/s11121-008-0093-x.
15. Stone, J., & Focella, E. (2011). Hypocrisy, dissonance and the self-regulation processes that improve health. Self and Identity, 10(3), 295-303. DOI: 10.1080/15298868.2010.538550
16. Stone, J. and Fernandez, N. C. (2008), To Practice What We Preach: The Use of Hypocrisy and Cognitive Dissonance to Motivate Behavior Change. Social and Personality Psychology Compass, 2: 1024–1051. doi: 10.1111/j.1751-9004.2008.00088.x
17. Freijy T1, Kothe EJ. Dissonance-based interventions for health behaviour change: a systematic review. Br J Health Psychol. 2013 May;18(2):310-37. doi: 10.1111/bjhp.12035.
18. Ahlwardt K, Heaivilin N, Gibbs J, Page J, Gerbert B, Tsoh JY. Tweeting about pain: comparing self-reported toothache experiences with those of backaches, earaches and headaches. J Am Dent Assoc. 2014 Jul;145(7):737-43. doi: 10.14219/jada.2014.30.
19. Bernardo TM, Rajic A, Young I, Robiadek K, Pham MT, Funk JA. Scoping review on search queries and social media for disease surveillance: a chronology of innovation. J Med Internet Res. 2013 Jul 18;15(7):e147. doi: 10.2196/jmir.2740.
20. Broniatowski DA, Paul MJ, Dredze M. National and local influenza surveillance through Twitter: an analysis of the 2012-2013 influenza epidemic. PLoS One. 2013 Dec 9;8(12):e83672. doi: 10.1371/journal.pone.0083672.
21. Integrating Social Media Monitoring Into Public Health Emergency Response Operations. Disaster Med Public Health Prep. 2016 May 27:1-6.
22. Thom D, Kruger R, Ertl T. Can Twitter Save Lives? A Broad-Scale Study on Visual Social Media Analytics for Public Safety. IEEE Trans Vis Comput Graph. 2016 Jul;22(7):1816-29. doi: 10.1109/TVCG.2015.2511733.
23. Chu KH, Allem JP, Cruz TB, Unger JB. Vaping on Instagram: cloud chasing, hand checks and product placement. Tob Control. 2016 Sep 22. pii: tobaccocontrol-2016-053052. doi: 10.1136/tobaccocontrol-2016-053052.

24. Kavuluru R, Sabbir AK. Toward automated e-cigarette surveillance: Spotting e-cigarette proponents on Twitter. J Biomed Inform. 2016 Jun;61:19-26. doi: 10.1016/j.jbi.2016.03.006
25. Kostygina G, Tran H, Shi Y, Kim Y, Emery S. ‘Sweeter Than a Swisher': amount and themes of little cigar and cigarillo content on Twitter. Tob Control. 2016 Oct;25(Suppl 1):i75-i82. doi: 10.1136/tobaccocontrol-2016-053094.
26. Freifeld CC, Brownstein JS, Menone CM, Bao W, Filice R, Kass-Hout T, Dasgupta N. Digital drug safety surveillance: monitoring pharmaceutical products in twitter. Drug Saf. 2014 May;37(5):343-50. doi: 10.1007/s40264-014-0155-x.
27. Correia RB, Li L, Rocha LM. Monitoring potential drug interactions and reactions via network analysis of Instagram user timelines. Pac Symp Biocomput. 2016;21:492-503.
28. Sarker A, Ginn R, Nikfarjam A, O'Connor K, Smith K, Jayaraman S, Upadhaya T, Gonzalez G. Utilizing social media data for pharmacovigilance: A review. J Biomed Inform. 2015 Apr;54:202-12. doi: 10.1016/j.jbi.2015.02.004. Epub 2015 Feb 23.
29. Adrover C, Bodnar T, Huang Z, Telenti A, Salathé M. Identifying Adverse Effects of HIV Drug Treatment and Associated Sentiments Using Twitter. JMIR Public Health Surveill. 2015 Jul 27;1(2):e7. doi: 10.2196/publichealth.4488.
30. Industry usage of social and digital media communities in clinical research. The Tufts Center for the Study of Drug Development. White Paper, June 2014.
31. Sugawara Y, Narimatsu H, Hozawa A, Shao L, Otani K, Fukao A. Cancer patients on Twitter: a novel patient community on social media. BMC Res Notes. 2012 Dec 27;5:699. doi: 10.1186/1756-0500-5-699.
32. Katz MS, Utengen A, Anderson PF, Thompson MA, Attai DJ, Johnston C, Dizon DS. Disease-Specific Hashtags for Online Communication About Cancer Care. JAMA Oncol. 2016 Mar;2(3):392-4. doi: 10.1001/jamaoncol.2015.3960.
33. Audun Utengen. The Rise of Patient Communities on Twitter – Twitter Visualized. Symplur blog. December, 2012. Web link: http://www.symplur.com/shorts/the-rise-of-patient-communities-on-twitter-visualized/
34. Rosenkrantz AB, Labib A, Pysarenko K, Prabhu V. What Do Patients Tweet About Their Mammography Experience? Acad Radiol. 2016 Sep 19. pii: S1076-6332(16)30175-1. doi: 10.1016/j.acra.2016.07.012.
35. Pinho-Costa L, Yakubu K, Hoedebecke K, Laranjo L, Patrick Reichel C, Colon-Gonzalez MD, Luísa Neves A, Errami H. Healthcare hashtag index development: Identifying global impact in social media. J Biomed Inform. 2016 Sep 16. pii: S1532-0464(16)30123-X. doi: 10.1016/j.jbi.2016.09.010.
36. Pemmaraju N, Utengen A, Gupta V, Kiladjian JJ, Mesa R, Thompson MA. Social Media and Myeloproliferative Neoplasms (MPN): Analysis of Advanced Metrics From the First Year of a New Twitter Community: #MPNSM. Curr Hematol Malig Rep. 2016 Aug 4.
37. Chiang AL, Vartabedian B, Spiegel B. Harnessing the Hashtag: A Standard Approach to GI Dialogue on Social Media. Am J Gastroenterol. 2016 Aug;111(8):1082-4. doi: 10.1038/ajg.2016.259.
38. Xu S, Markson C, Costello KL, Xing CY, Demissie K, Llanos AA. Leveraging Social Media to Promote Public Health Knowledge: Example of Cancer Awareness via Twitter. JMIR Public Health Surveill. 2016 Apr 28;2(1):e17. Doi: 10.2196/publichealth.5205.
39. Sedrak MS, Cohen RB, Merchant RM, Schapira MM. Cancer Communication in the Social Media Age. JAMA Oncol. 2016;2(6):822-823. Doi:10.1001/jamaoncol.2015.5475.
40. Harris PA Taylor R, Thielke R, Payne J, Gonzalez N, Conde JG. Research electronic data capture (REDCap) - a metadata-driven methodology and workflow process for providing translational research informatics support. J Biomed Inform. 2009;42(2):377-381.

41. IBM Corp. Released 2015. IBM SPSS Statistics for Mac, Version 22.0. Armonk, NY: IBM Corp.
42. Report on clinical trial information seekers. Perceptions and insights study. The Center for Information & Study on Clinical Research Participation; 2013.
43. Harris-Interactive, 2004.
44. Report on clinical trial information seekers. Perceptions and insights study. The Center for Information & Study on Clinical Research Participation; 2013.
45. Smith, A. Older adults and technology use. Pew Research Center. http://www.pewinternet.org/2014/04/03/older-adults-and-technology-use/.
46. Jens Manuel Krogstad. Social media preferences vary by race and ethnicity. Pew Research, 2015. Web link: http://www.pewresearch.org/fact-tank/2015/02/03/social-media-preferences-vary-by-race-and-ethnicity/.
47. Young African Americans have high levels of Twitter use. Pew Research, 2014. Web link: http://www.pewinternet.org/2014/01/06/african-americans-and-technology-use/young-african-americans-have-high-levels-of-twitter-use/
48. VI. Social Networking. Mark Hugo Lopez, Ana Gonzalez-Barrera, Eileen Patten. Pew Research, 2013. Web link: http://www.pewhispanic.org/2013/03/07/vi-social-networking/
49. Social Media Usage: 2005-2015. Pew Research, Oct 2015. Web lik: http://www.pewinternet.org/2015/10/08/social-networking-usage-2005-2015/
50. Boyd, D., & Crawford, K. (2012). Critical questions for big data. Information, Communication & Society, 15(5), 662-679.
51. Bruns, A., Burgess, J., Highfield, T., Kirchhoff, L., & Nicolai, T. (2011). Mapping the Australian networked public sphere. Social Science Computer Review, 29(3), 277- 287.
52. Bekkers V, Edwards A, de Kool D. Social media monitoring: Responsive governance in the shadow of surveillance? Government Information Quarterly 30 (2013) 335–342.
53. Bender JL, Cyr AB, Arbuckle L, Ferris LE. Ethics and Privacy Implications of Using the Internet and Social Media to Recruit Participants for Health Research: A Privacy-by-Design Framework for Online Recruitment. J Med Internet Res 2017;19(4):e104 DOI: 10.2196/jmir.7029.
54. Gelinas L, Pierce R, Winkler S, Cohen IG, Lynch HF, Bierer BE. Using Social Media as a Research Recruitment Tool: Ethical Issues and Recommendations. Am J Bioeth. 2017 Mar;17(3):3-14. doi: 10.1080/15265161.2016.1276644.

APPENDIX 1: FULL TWITTER USER DATA ANALYSIS BY CANCER DISEASE TYPE IN CALIFORNIA

English Twitter messages, Analysis period: Jan 1, 2016 - Jan 30, 2017

Disease	Users	Tweets	Source: Symplur Signals
Breast Cancer	14,782	76,952	https://signals.symplur.com/r/608dc26cdd
Leukemia	2,645	13,166	https://signals.symplur.com/r/5d7c16e238
Lung Cancer	3,107	16,106	https://signals.symplur.com/r/c816c460c1
Pancreatic Cancer	1,269	5,345	https://signals.symplur.com/r/3a15039a1a
Brain Cancer	1,741	4,365	https://signals.symplur.com/r/e2b3f1bb35
Sarcoma	257	431	https://signals.symplur.com/r/5d64e7caac
Kidney Cancer	690	1,850	https://signals.symplur.com/r/9d4aa2b40a
Colon Cancer	1,882	5,585	https://signals.symplur.com/r/e07fac2dcd
Head & Neck Cancer	102	398	https://signals.symplur.com/r/249ec3b4f2
Gynecological Cancer	191	1,124	https://signals.symplur.com/r/4881c25b33
Prostate Cancer	4,610	12,950	https://signals.symplur.com/r/20c3f0ca66
Skin Cancer	2,298	8,108	https://signals.symplur.com/r/f913742b24
Liver Cancer	194	315	https://signals.symplur.com/r/f33ba92527
Bladder Cancer	306	751	https://signals.symplur.com/r/52bb7efe93
Lymphoma	1,445	8,711	https://signals.symplur.com/r/f986385f5b
Myeloma	983	3,239	https://signals.symplur.com/r/b04cc2742f

APPENDIX 2: ACCRUALS and ACCRUAL TARGETS BY TUMOR TYPE

Lung, Colon, Breast, Prostate, Kidney and Lymphoma

Patient Accrual Diagnosis Site: Lung						
**Trial Category**	**2017**	**2016**	**2015**	**2014**	**2013**	**2012**
**Interventional Therapeutic**	17	33	34	19	16	32
**Interventional (Therapeutic and Non-Therapeutic)**	19	37	34	19	16	32

*2017 accruals are from 1/1/2017 to 06/27/2017.

Patient Accrual Diagnosis Site: Colon						
**Trial Category**	**2017**	**2016**	**2015**	**2014**	**2013**	**2012**
**Interventional Therapeutic**	21	49	83	81	45	47
**Interventional (Therapeutic and Non-Therapeutic)**	21	49	83	81	46	47

*2017 accruals are from 1/1/2017 to 06/27/2017.

Patient Accrual Diagnosis Site: Breast						
**Trial Category**	**2017**	**2016**	**2015**	**2014**	**2013**	**2012**
**Interventional Therapeutic**	33	64	63	79	104	151
**Interventional (Therapeutic and Non-Therapeutic)**	35	75	117	167	162	211

*2017 accruals are from 1/1/2017 to 06/27/2017.

Patient Accrual Diagnosis Site: Prostate						
**Trial Category**	**2017**	**2016**	**2015**	**2014**	**2013**	**2012**
**Interventional Therapeutic**	21	52	68	69	68	58
**Interventional (Therapeutic and Non-Therapeutic)**	23	52	68	71	68	79

*2017 accruals are from 1/1/2017 to 06/27/2017.

Patient Accrual Diagnosis Site: Kidney						
**Trial Category**	**2017**	**2016**	**2015**	**2014**	**2013**	**2012**
**Interventional Therapeutic**	4	7	27	49	36	30
**Interventional (Therapeutic and Non-Therapeutic)**	4	7	27	49	36	30

*2017 accruals are from 1/1/2017 to 06/27/2017.

Patient Accrual Diagnosis Site: Lymphoma						
**Trial Category**	**2017**	**2016**	**2015**	**2014**	**2013**	**2012**
**Interventional Therapeutic**	4	9	7	8	11	12
**Interventional (Therapeutic and Non-Therapeutic)**	4	9	7	8	11	12

*2017 accruals are from 1/1/2017 to 06/27/2017.

APPENDIX 3: Overview of Twitter Outreach Message Templates

Twitter recruitment message templates we propose to use for contacting those Twitter users in LA County

(i.e., those who mentioned one of the select disease categories lung cancer, colon cancer, breast cancer, prostate cancer, kidney cancer and lymphoma in the past 6 months or during the research study period)

Abbreviation: URL = Uniform Resource Identifier is the global address of documents and other resources on the World Wide Web. The URL to the respective trial group will be included in each Twitter recruitment message. For example, a message about open lung cancer trials at USC Norris will link to the web page that lists all lung cancer trials.

#disease will be replaced by the respective disease.

INITIAL CONTACT

Dear @JaneDoe or Hi @JaneDoe or Hello @JaneDoe,

- We noticed your interest in <u>#disease</u> and wanted to share the latest open clinical research opportunities @KeckMedUSC. You can find more information here: URL #ClinicalTrial
- We noticed your mention of <u>#disease</u> and wanted to reach out. Did you know about these open <u>#disease</u> studies @KeckMedUSC? You can find more information here: URL #ClinicalTrial
- We noticed your interest in <u>#disease</u> and wanted to share the latest open clinical research opportunities @KeckMedUSC. You can find more information here: URL #ClinicalTrial
- We noticed your interest in <u>#disease</u> and wanted to share that the following <u>#disease</u> clinical trials @KeckMedUSC are looking for participants. More information is available here: URL #ClinicalTrial
- We noticed your interest in <u>#disease</u> and thought you might be interested in open <u>#disease</u> clinical trials @KeckMedUSC that are looking for participants. More information is available here: URL #ClinicalTrial

FOLLOW-UP IF NO RESPONSE AFTER INITIAL CONTACT

If a prospective participant does not respond, we will send a <u>one-time follow-up message</u> within two weeks of initial contacting.

Dear @JaneDoe or Hi @JaneDoe,

- Let us know if you have any questions about open #disease trials @KeckMedUSC. We'd be happy to help. Feel free to send us a direct message.

- We're following up on our previous message about clinical trials @KeckMedUSC. Feel free to contact us via DM if you have any questions. We'd be happy to help.
- Let us know via direct message if you have any questions about #disease trials @KeckMedUSC. We'd be happy to help.
- Did you have any questions about ongoing #disease trials @KeckMedUSC? Let us know. Feel free to contact us via DM. We'd be happy to help.

Twitter message templates we propose to use to manage data security and privacy issues

Dear @JaneDoe: To protect your privacy, we suggest you delete your message. Please contact us directly URL

Hi @JaneDoe: The security of social media is not guaranteed. Contact us about the study. Don't post if concerned about privacy.

Message templates to convey general information about participating in clinical trials and this particular project

The following content is based on educational information developed by the National Institutes of Health (NIH) for the initiative "NIH clinical research trials and you" (https://www.nih.gov/health-information/nih-clinical-research-trials-you)

Note: Study team members Dr. Reuter and Namquyen Le will take any resulting phone calls and triage them as needed.

Project

We're part of a research project aimed at understanding if Twitter can be used to better connect patients with clinical research opportunities.

We're reaching out to you as part of a research project trying to understand if Twitter can be used to better connect patients with clinical research opportunities.

Clinical trials

It's your involvement that helps researchers to uncover better ways to prevent, diagnose, & treat diseases. Participation is always voluntary and your choice.

Clinical trials are part of clinical research and at the heart of all medical advances. Participation is always voluntary and your choice.

Clinical trials look at new ways to prevent, detect, or treat disease. Participation is your choice and always voluntary.

The goal of clinical trials is to determine if a new test or treatment works and is safe. Participation is your choice and always voluntary.

People participate in clinical trials for a variety of reasons. Participation is your choice and always voluntary.

Clinical trials offer the possibility to have the additional care and attention from the clinical trial staff. Participation is your choice and always voluntary.

APPENDIX 4: Study Web Page Example

The screenshot below shows an example of a study page in the USC Clinical Studies Directory. The study web pages used in this research project will use the same type of template but will differ in that they will have a disease specific parent page that lists all recruiting trials enrolled in this study – one parent page for each selected cancer type: lung cancer, colon cancer, breast cancer, prostate cancer, kidney cancer and lymphoma. For example, all recruiting lung cancer studies enrolled in this study will be listed on this parent page and have individual pages as shown below.

The easy-to-use contact form asks for the name and email of a user and triggers an email to the SC CTSI team (Dr. Reuter and Namquyen Le) who will triage the request to the correct disease contact at USC Norris. The web page content used for this research will be submitted to the IRB once we enroll the trials and know their focus and consent information. The consent information will guide the content we use on the web pages.

Example of a clinical study web page in the USC Clinical Studies Directory

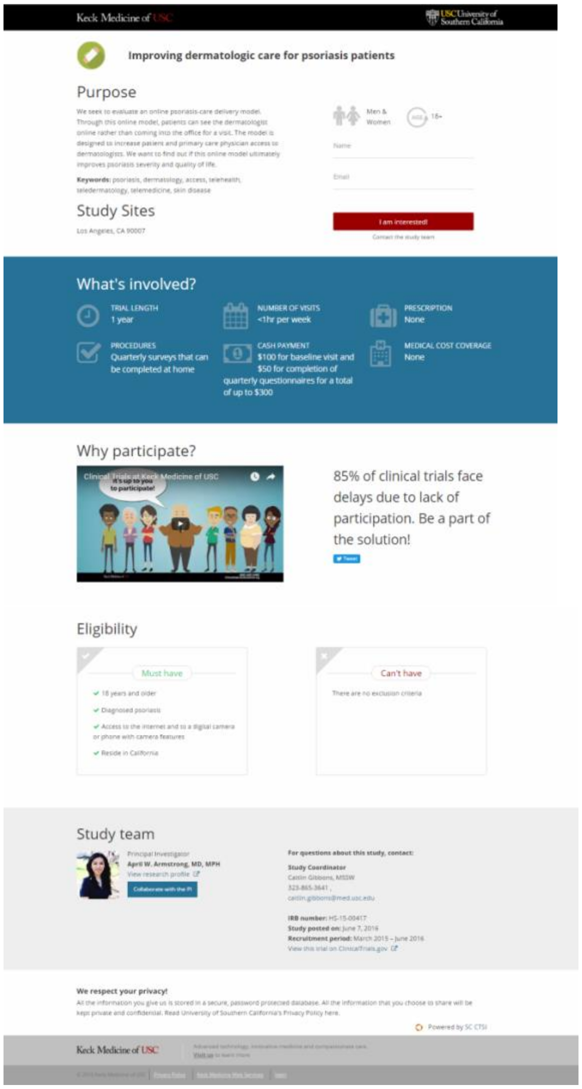
Keck Medicine of USC
USC University of Southern California
Improving dermatologic care for psoriasis patients
Purpose
We seek to evaluate an online psoriasis-care delivery model. Through this online model, patients can see the dermatologist online rather than coming into the office for a visit. The model is designed to increase patient and primary care physician access to dermatologists. We want to find out if this online model ultimately improves psoriasis severity and quality of life.
Keywords: psoriasis, dermatology, access, telehealth, teledermatology, telemedicine, skin disease
Men & Women
18+
Name
Email
I am interested!
Contact the study team
Study Sites
Los Angeles, CA 90007
What's involved?
TRIAL LENGTH
1 year
NUMBER OF VISITS
<1hr per week
PRESCRIPTION
None
PROCEDURES
Quarterly surveys that can be completed at home
CASH PAYMENT
$100 for baseline visit and $50 for completion of quarterly questionnaires for a total of up to $300
MEDICAL COST COVERAGE
None
Why participate?
Clinical Trials at Keck Medicine of USC
It's up to you to participate!
85% of clinical trials face delays due to lack of participation. Be a part of the solution!
Eligibility
Must have
18 years and older
Diagnosed psoriasis
Access to the internet and to a digital camera or phone with camera features
Reside in California
Can't have
There are no exclusion criteria
Study team
Principal Investigator
April W. Armstrong, MD, MPH
View research profile
Collaborate with the PI
For questions about this study, contact:
Study Coordinator
Caitlin Gibbons, MSSW
323-865-3641 ,
caitlin.gibbons@med.usc.edu
IRB number: HS-15-00417
Study posted on: June 7, 2016
Recruitment period: March 2015 – June 2016
View this trial on ClinicalTrials.gov
We respect your privacy!
All the information you give us is stored in a secure, password protected database. All the information that you choose to share will be kept private and confidential. Read University of Southern California's Privacy Policy here.
Powered by SC CTSI
Keck Medicine of USC

APPENDIX 5. DATA DICTIONARY WITH ALL MEASURES OF SUCCESS USED IN THIS RESEARCH PROJECT

Measurement	Variable name	Type of variable	Variable label	Value labels	Comments
Surveillance data	sm_topic	Text	Disease topic	1=non-small cell lung cancer 2=colon cancer 3=breast cancer 4=prostate cancer 5=kidney cancer 6=lymphoma	
Surveillance data	user_name	Text	User Twitter name		
Surveillance data	user_description	Text	User Twitter description		
Surveillance data	user_location	Text	User Twitter location		
Surveillance data	twitter_message	Text	Twitter message that mentions any of the targeted disease terms		
Surveillance data	tate_sent	Numeric	Date of Twitter user message		mm/dd/yyyy
Contact data	contact_message	Text	Content of contact message		
Contact data	date_sent	Numeric	Date of contact message		mm/dd/yyyy
Contact data	sol_acknowledgement	Numeric	Contact acknowledged solicitation	1=yes 0=no	
Contact data	date_acknowledgement	Numeric	Date contact acknowledged solicitation		mm/dd/yyyy
Contact data	patient	Numeric	Is the contacted person a cancer patient?	1=yes 0=no	
Contact data	patient_cancer_type	Text	Type of cancer contact was diagnosed with	1=non-small cell lung cancer 2=colon cancer 3=breast cancer 4=prostate cancer 5=kidney cancer 6=lymphoma 7=Other	
Contact data	eligibility	Numeric	Is the contacted person eligible for one of the trials?	1=yes 0=no	
Contact data	first_name	Text	Contact's first name		
Contact data	last_name	Text	Contact's last name		
Contact data	gender	Numeric	Gender	1=Male 2=Female	
Contact data	race/ethnicity	Numeric	Race/ethnicity	1=African American / Black 2=American Indian / Alaska Native 3=Asian / Pacific Islander 4=Hispanic 5=Middle Eastern 6=White 7=Other	

Contact data	Education	Numeric	Highest education	1=No schooling completed 2=Nursery school to 8th grade 3=Some high school, no diploma 4=High school graduate, diploma or the equivalent (for example: GED) 5=Some college credit, no degree 6=Trade/technical/vocational training 7=Associate degree 8=Bachelor's degree 9=Master's degree 10=Professional degree 11=Doctorate degree	
Contact data	intervention-response	Numeric	Did you respond to the social media recruitment message because you would like to learn more about a clinical trial?	1=yes 0=no	
Contact data	info_purpose	Numeric	Are you gathering information for yourself or on behalf of someone else?	1=For myself 2=On behalf of someone else	
Contact data	value	Numeric	How helpful would you say is it when research institutions reach out directly to potential clinical trial participants via Twitter?	1=Very helpful 2=Helpful 3=Somewhat helpful 4=Not helpful at all 5=Don't know	
Contact data	concern	Numeric	How concerned are you about researchers and research institutions monitoring Twitter conversations to identify and contact potential study participants for clinical trials?	1=Very concerned 2=Somewhat concerned 3=Not too concerned 4=Not concerned at all 5=Don't know	
Contact data	contact_form_use	Numeric	Contact used contact form on clinical trial web page	1=yes 0=no	
Contact data	follow-up	Numeric	USC Norris team followed up with contact	1=yes 0=no	
Contact data	consultation	Numeric	Contact had formal consultation with a USC/Norris physician or trial coordinator	1=yes 0=no	
Contact data	enrolled	Numeric	Contact enrolled	1=yes 0=no	
Message data	day-sent	Numeric	Day message was sent	1=Monday 2=Tuesday 3=Wednesday 4=Thursday 5=Friday 6=Saturday 7=Sunday	
Message data	time_sent	Numeric	Time message was sent	hh:mm:sec	Not available for ads since social media networks have an internal process for displaying ads.

Message data	image	Numeric	Image included in the message	0=No 1=Yes	
Message data	message_clicks	Numeric	Total number of message link clicks after 1 week	0, 1, 2, 3,…	
Message data	click_time	Numeric	Time of click on message link		hh:mm:sec (Data will be collected using two approaches (1) using third-party application ClickMeter and (B) rails backend (ie, when a webpage is loaded on the educational website itself. The SM platforms and Google don't provide this type of information.)
Message data	impressions	Numeric	Total number of message impressions after 1 week	0, 1, 2, 3,…	
Message data	retweet_Twitter	Numeric	Message shares	0, 1, 2, 3,…	
Message data	reply_Twitter	Numeric	Message replies/comments	0, 1, 2, 3,…	
Message data	likes_Twitter	Numeric	Message likes	0, 1, 2, 3,…	
Website data	sessions	Numeric	Website sessions referred to by Twitter message after 1 week	0, 1, 2, 3,…	
Website data	clicks	Numeric	Website link clicks	0, 1, 2, 3,…	
Website data	users	Numeric	Website users	0, 1, 2, 3,…	
Website data	exits	Numeric	Webisite exits	0, 1, 2, 3,…	
Website data	session_duration	Numeric	Duration (in seconds) of users' sessions.	0, 1, 2, 3,…	
Website data	time_onpage	Numeric	Time on page	0, 1, 2, 3,…	
Website data	pageviews	Numeric	Pageviews	0, 1, 2, 3,…	

APPENDIX 6. QUESTIONNAIRE FOR PROSPECTIVE STUDY PARTICIPANTS

(i.e., those who contacted the study team in response to the SM recruitment)

1. Prospective participant survey consent (oral, over the phone)

Are you 18 or older?

- Yes (proceed)
- No (We are sorry but participants must be 18 or older to participate. We appreciate your interest.)

Purpose of the study: We are studying the acceptability and effectiveness of social media listening and recruitment on Twitter to enhance enrollment for clinical trials. Your participation in this 5-min survey will help us understand who we were able to reach with these messages, and allow us to connect you with the right team at the USC Norris Cancer Center. The questions will ask demographic information, such as your age, gender, education, and your opinion about the use of Twitter, in particular listening to Twitter user conversations, for clinical trial recruitment.

Voluntary participation: Participating in this survey is entirely voluntary and anonymous. You can withdraw from the survey or skip questions at any time without negative consequences. The information you provide will be confidential and only used for the purpose of this research project. Your information will not be shared with third parties.

Cost: There is no cost to you for taking part in this study and completing this survey.

Compensation: There is no compensation for taking part in this study and completing this survey.

If you have further questions, feel free to contact the study team at the University of Southern California. Principal investigator: Katja Reuter (katja.reuter@usc.edu).

Do you consent?

- Agree
- Don’t agree.

---

2. Prospective participant data collection sheet

1. Name of prospective participant
    a. First name
    b. Last name

2. What's your Twitter handle?

    a. ________________________
    b. Prefer not to disclose

3. What's your email?

4. What's your birth date

5. Are you…?
    - Female
    - Male
    - Other
    - Prefer not to disclose

6. How do you describe yourself? (check all that apply)
    - African American / Black
    - American Indian / Alaska Native
    - Asian / Pacific Islander
    - Hispanic
    - Middle Eastern
    - White
    - Other
    - Prefer not to disclose

7. What is the highest degree or level of school you have completed?
    - No schooling completed
    - Nursery school to 8th grade
    - Some high school, no diploma
    - High school graduate, diploma or the equivalent (for example: GED)
    - Some college credit, no degree
    - Trade/technical/vocational training
    - Associate degree
    - Bachelor's degree
    - Master's degree
    - Professional degree
    - Doctorate degree
    - Prefer not to disclose

8. Did you respond to the social media recruitment message because you would like to learn more about a clinical trial?
    a. Yes
    b. No

9. Are you gathering information for yourself or on behalf of someone else?
    a. For myself
    b. On behalf of someone else

10. How helpful would you say is it when research institutions reach out directly to potential clinical trial participants via Twitter?
    a. Very helpful
    b. Helpful
    c. Somewhat helpful
    d. Not helpful at all
    e. Don't know

11. How concerned are you about researchers and research institutions monitoring Twitter conversations to identify and contact potential study participants for clinical trials?
    a. Very concerned
    b. Somewhat concerned
    c. Not too concerned
    d. Not concerned at all
    e. Don't know

12. When were you diagnosed with cancer?

13. Do you have active cancer? (Only if yes triage to USC Norris team)
    a. Is it visible on scans (CT, MRI)?
    b. Are you on active treatment?

14. Are you able to do activities of daily life independently? (e.g., eating, drinking, bathing)

15. Do you have a copy of your medical records?

16. Are you currently a patient at
    a. USC Norris Comprehensive Cancer Center, or
    b. USC Keck Medical Center, or
    c. LA County USC?

17. Any other comments you want to share (free text)
