# Supplementary material for: Monitoring Twitter Conversations for Targeted Recruitment in Cancer Trials in Los Angeles County: Protocol for a Mixed-Methods Pilot Study
Source: JMIR Res Protoc. 2018 Sep 25;7(9):e177. doi: 10.2196/resprot.9762 (PMC6231794; doi:10.2196/resprot.9762)
Supplement: Multimedia Appendix 2 [file resprot_v7i9e177_app2.pdf]

Clinical Investigations Committee
Memorandum

To: Thomas A. Buchanan, MD

From: Syma Iqbal, MD
Co-Chair, Clinical Investigations Committee

Zeno Ashai
Administrator, Clinical Investigations Committee
Assistant Director, CISO

Date: September 6, 2017

Re: **CIC Review of Protocol: 0S-17-7** The Use of Social Media Listening for Targeted Recruitment of Twitter users in LA County in Cancer Trials Compared to Historic Recruitment Data: A Mixed-Methods Study

Your protocol was reviewed by the CIC on September 6, 2017 and received contingent approval

**Priority Score: 2.4**	
Outstanding	***1.0-1.5***
Excellent	***1.5-2.0***
Very Good	***2.0-2.5***
Good	***2.5-3.5***
Acceptable	***3.5-5.0***

The study received contingent approval

The following materials were reviewed by the Clinical Investigations Committee:

1. CIC Protocol Submission Checklist
2. Protocol (08/21/2017)

Competing Trials: None

Summary:
This is a USC investigator initiated trial. There is a significant need to improve enrollment to cancer clinical trials (a significant number do not meet accrual goals) this is an interesting attempt to increase recruitment to clinical trials by leveraging social media, in this case Twitter. The investigators will use a commercial aggregator of Twitter traffic to identify potential patients who may be interested in clinical trial participation. The two overall objectives are (1) to assess acceptance among those managing trials in this mode of recruitment, and (2) to assess the impact of this approach on recruitment to targeted trials. This study will examine the feasibility and gain preliminary data on the impact of targeted social media listening (SML) as a tool for enhancing recruitment to cancer trials (non-small cell lung cancer, colon cancer, breast cancer, prostate cancer, kidney cancer, lymphoma) among Twitter users in LA County. Targeted SML employs community-generated data from Twitter to identify prospective participants based on health interests and disease topics they mention in their Twitter messages. Those individuals are then targeted for recruitment using a combination of SM and traditional methods. Success will be compared to historical success achieved without the use of SML.

This is a well written and very interesting study, however, few clarifications are required prior to final approval.

The committee voted for contingent approval.

Risk Category: "Low Risk" requiring Annual review.

The items requiring a written response are as follows:

1. Given the many thousands of twitter disease-related messages and their lack of specificity, if all these twitter users are automatically contacted via twitter and even a small fraction of them responds, could it not overwhelm the disease-group specialists tasked with contacting responders to assess eligibility? If so, how will this be addressed? There is no specific plan provided.

2. ***** The investigators should describe in detail how they intend to characterize the success of the individual contacts that they make through twitter, and collect the specific outcome measures describe below.

3. The investigator should describe the mechanism they will use to identify whether an enrollee on a USC/Norris cancer trial had been contacted through the investigators' program.

***** The primary assessment of efficacy is based on a comparison with historical recruitment statistics. Over the short 9 month run of this intervention, it will be difficult to ascribe any changes in the very low monthly recruitment rates of the target diagnoses to this intervention *vs.* other external influences or just usual Poisson variation. There also is no attempt that I can see to differentiate between enrollees who have been contacted via this intervention vs those who come in through regular channels.

It would seem to be very important to collect statistics on the outcome of contacts. The investigators will be direct-messaging individuals who have mentioned cancer. Thus, one would want to collect data on the following, which would provide a direct measure of the yield of this effort, but none of this is described in the protocol.

- Number of contacts solicited
- Number of contacts who acknowledge the solicitation
- Numbers of contracts who are the principals for cancer (i.e. have been diagnosed) or who have direct influence on the principal for cancer.
- How many of these principal individuals are potentially eligible for a clinical trials at USC/Norris when they are contacted (e.g., they are at a place during their diagnosis that they could consider a clinical trial).
- How many of these principals had a formal consultation with a USC/Norris physician or trial coordinator.
- How many were enrolled on a clinical trial.

Note to PI: Response must include a cover letter addressing each of the stipulations/clarifications point by point using the same number sequence. Revisions must be indicated by underlining added text and striking through deleted text. Response should be submitted to the CIC@med.usc.edu and zeno.ashai@med.usc.edu.

To: Syma Iqbal, M.D.
Co-Chair, Clinical Investigations Committee

From: Thomas A. Buchanan, M.D. (PI) and Katja Reuter, PhD (Co-PI, contact PI)

Date: September 26, 2017

Re: Response to CIC Review of Protocol: 0S-17-7

THE USE OF SOCIAL MEDIA LISTENING FOR TARGETED RECRUITMENT OF TWITTER USERS IN LA COUNTY IN CANCER TRIALS COMPARED TO HISTORIC RECRUITMENT DATA: A MIXED- METHODS STUDY

Thank you very much for your timely and detailed review of 0S-17-7. Below you will find our responses to the CIC comments.

1. Given the many thousands of Twitter disease-related messages and their lack of specificity, if all these Twitter users are automatically contacted via Twitter and even a small fraction of them responds, could it not overwhelm the disease-group specialists tasked with contacting responders to assess eligibility? If so, how will this be addressed? There is no specific plan provided.

Per section 5.2 "Anticipated challenges" under scientific issues, item "Managing volume of incoming requests through clinical trial web pages," we would like to provide more information to clarify this point. This project will target Twitter users who live in the Los Angeles area as indicated by self-reported data from Twitter. Since this is a pilot study and no data exist to estimate the volume of contact requests via Twitter, the first point of contact will be the main SC CTSI study team (Dr. Reuter and Namquyen Le). This way, the SC CTSI team will be able to triage only relevant requests to the respective disease group at USC Norris. In case of high volume, we will work closely with the affected disease group team at USC Norris to assist with pre-screening of requests and prospective participants. We added the following information to the protocol: "More specifically, we will add a pre-screening form to the respective clinical trial disease web page that will allow prospective participants to self-report key eligibility-related questions. This information will help the study team to determine eligibility and next steps. The system developed by SC CTSI will automatically classify responses as concordant, discordant, or neutral for study eligibility. This approach will allow us to extend the capacity of the involved study team members by prioritizing and focusing enrollment efforts on those individuals that may be most likely to meet final eligibility criteria."

2. * The investigators should describe in detail how they intend to characterize the success of the individual contacts that they make through Twitter, and collect the specific outcome measures described below.

We would like to provide more information about the complete set of metrics we will use for characterizing the success of all aspects of the intervention. We included a data dictionary (Appendix 5) in the protocol (referenced in section 3.3.7) that outlines the full list of metrics we intend to use, including additional metrics suggested by the reviewers. We added the following text to the proposal section 3.3.7 "Table 3 lists all primary outcomes measures we intend to use. In addition, Appendix 5 (data dictionary) provides the full set of metrics we will use to assess the success of the intervention, which includes additional success metrics such as number of contacts solicited, number of contacts who acknowledge the solicitation, number of social media contact/recruitment messages sent, day/time of contact, day/time of response."

Appendix 5 also outlines the information we will collect about prospective participants and enrollees who have been contacted via the Twitter intervention. This information is critical differentiate between enrollees who have been contacted via this intervention vs. those who come in through regular channels. We added additional information to the protocol in section 5.2 "Anticipated challenges", Table 4 under "scientific challenges" to clarify this point. We added the following clarification: "Our data collection approach will allow us to differentiate between enrollees who have been contacted via the Twitter intervention vs. those who come in through regular channels. First, we will collect publically available information about the Twitter users including their Twitter name, location, and Twitter account description. Second, we will correlate this information where possible with the name and email information users provide on the clinical trial web pages used in this research project. At this point, the SC CTSI team will triage the contact request to the USC Norris team that will contact the prospective trial participant to perform the eligibility screening. The USC Norris team will be provided with a tracking sheet to track additional information about the prospective participant including their Twitter name (if they came through Twitter). This will allow us to link up the initial contact data with the enrollee data and to differentiate between enrollees who have been contacted via the Twitter intervention vs. those who come in through regular channels. See also Appendix 5, an overview of the success metrics used for data collection."

3. The investigator should describe the mechanism they will use to identify whether an enrollee on a USC/Norris cancer trial had been contacted through the investigators' program.

The study teams will use tracking sheets to do so. We added more information to protocol section 3.3.5 to address this concern: "The study teams at SC CTSI and USC Norris will be provided with tracking sheets to collect data on the prospective participants and enrollees. For example, the SC CTSI team will track information on who was contacted via Twitter, when a Twitter user was contacted, their online engagement with the recruitment message(s), and if they used the contact form on the clinical trial web page to contact the study team. The USC Norris team will use a tracking sheet to track information about the prospective participants that were screened, their response, and whether an enrollee of a USC Norris cancer trial had been contacted through the investigators' program. Please see Table 3 for an overview of all

primary outcome measures and Appendix 5 for the data dictionary that list all metrics the study team will use to characterize the success of the intervention."

*****The primary assessment of efficacy is based on a comparison with historical recruitment statistics. Over the short 9 month run of this intervention, it will be difficult to ascribe any changes in the very low monthly recruitment rates of the target diagnoses to this intervention vs. other external influences or just usual Poisson variation. There also is no attempt that I can see to differentiate between enrollees who have been contacted via this intervention vs. those who come in through regular channels.

It would seem to be very important to collect statistics on the outcome of contacts. The investigators will be direct-messaging individuals who have mentioned cancer. Thus, one would want to collect data on the following, which would provide a direct measure of the yield of this effort, but none of this is described in the protocol.

- Number of contacts solicited
- Number of contacts who acknowledge the solicitation
- Numbers of contacts who are the principals for cancer (i.e. have been diagnosed) or who have direct influence on the principal for cancer.
- How many of these principal individuals are potentially eligible for a clinical trial at USC/Norris when they are contacted (e.g., they are at a place during their diagnosis that they could consider a clinical trial).
- How many of these principals had a formal consultation with a USC/Norris physician or trial coordinator. How many were enrolled on a clinical trial

CLINICAL INVESTIGATIONS SUPPORT OFFICE

USC/Norris Comprehensive Cancer Center
1441 Eastlake Ave. Room 7310E
Los Angeles, CA 90033

Date: October 3, 2017

To: Syma Iqbal, MD

Please review the responses provided by Dr. Buchanan for protocol 0S-17-7 reviewed by CIC on September 6, 2017

The following documents are enclosed:

1. CIC memo
2. PI response to CIC stipulation
3. Initial CIC package
4. Updated protocol
5. Designated reviewer approvals

Thank you,

Zeno Ashai

[✓] Approved

[ ] Not approved

Comments:

Signature: [signature]　　　　Date: 10/3/17

/mr
