# Supplementary material for: Monitoring Twitter Conversations for Targeted Recruitment in Cancer Trials in Los Angeles County: Protocol for a Mixed-Methods Pilot Study
Source: JMIR Res Protoc. 2018 Sep 25;7(9):e177. doi: 10.2196/resprot.9762 (PMC6231794; doi:10.2196/resprot.9762)
Supplement: Multimedia Appendix 3 [file resprot_v7i9e177_app3.pdf]

USC Children's Hospital Los Angeles LAC+USC iStar

Proposal #HS-17-00811

University of Southern California Health Sciences Campus
Institutional Review Board
LAC+USC Medical Center, General Hospital Suite 4700
1200 North State Street, Los Angeles, CA 90033
(323) 223-2340 phone
(323) 224-8389 fax
irb@usc.edu

Date: Oct 23, 2017, 09:00am

To: Thomas Buchanan, M.D.
ENDOCRINOLOGY AND DIABETES
Daisy Sosa, BA
Data Manager
CLINICAL INVESTIGATIONS SUPPORT OFFICE (CISO)

From: Health Sciences Institutional Review Board
General Hospital, Suite 4700
1200 North State Street
Los Angeles, CA 90033
(323) 223-2340

TITLE OF PROPOSAL:
0S-17-7: The use of social media listening for targeted recruitment of Twitter users in LA County in cancer trials compared to historic recruitment data: A mixed-methods study (0S-17-7: Use of social media for recruitment in LA County in cancer trials compare to historic recruitment)

Action Date: **10/23/2017** Action Taken: **Approve**

Committee: Institutional Review Board Chairman

Note: Your iStar application and attachments were reviewed by the expedited mechanism by Dr. Deirdre Anglin on October 23, 2017.

The project was APPROVED.

The materials submitted and considered for review of this project included:
1. iStar application, dated 10/12/2017
2. Protocol, dated 10/02/2017

3. Questionnaire, dated 10/02/2017
4. Twitter Recruitment Text, dated 10/02/2017
5. Verbal Script, dated 10/12/2017

Approval of your study will expire at the end of the day (midnight) on October 22, 2018.

Based on the information submitted for review, this study qualifies for expedited review according to §46.110(b) (7).

In approving this research, the IRB determined that all of the following requirements (45CFR 46.111) were satisfied: (1) Risks to subjects are minimized: (i) by using procedures which are consistent with sound research design and which do not unnecessarily expose subjects to risk, and (ii) whenever appropriate, by using procedures already being performed on the subjects for diagnostic or treatment purposes. (2) Risks to subjects are reasonable in relation to anticipated benefits, if any, to subjects, and the importance of the knowledge that may reasonably be expected to result. In evaluating risks and benefits, only those risks and benefits that may result from the research are considered (as distinguished from risks and benefits of therapies subjects would receive even if not participating in the research). (3) Selection of subjects is equitable (the purposes of the research and the setting in which the research will be conducted were taken into account). (4) Informed consent will be sought from each prospective subject or the subject's legally authorized representative, in accordance with, and to the extent required by 45CFR 46.116. (5) Informed consent will be appropriately documented, in accordance with, and to the extent required by 45CFR 46.117. (6) When appropriate, the research plan makes adequate provision for monitoring the data collected to ensure the safety of subjects. (7) When appropriate, there are adequate provisions to protect the privacy of subjects and to maintain the confidentiality of data.

As the Principal Investigator you are required to ensure that this research and the actions of all project personnel involved in conducting the study will conform with the research project and its modifications approved by the IRB; HHS regulations (45CFR46); FDA regulations (21CFR50,56); International Conference on Harmonization Good Clinical Practice Consolidated Guideline; IRB Policies and Procedures and applicable state laws. Failure to comply may result in suspension or termination of your research project, notification of appropriate governmental agencies by the IRB, and/or suspension of your freedom to present or publish results. Any proposed changes in the research project must be submitted, reviewed and approved by the IRB before the change can be implemented. The only exception is a change necessary to eliminate apparent immediate hazards to the research subjects. In such a case, the IRB should be promptly informed of the change following its implementation for IRB review. You must inform the IRB immediately if you become aware of any violations of HHS regulations (45CFR46), FDA regulations (21CFR50,56), applicable state laws or IRB Policies and Procedures for the protection of human subjects. You are required to notify the IRB office in the event of any action by the sponsor, funding agency or FDA, including warnings, suspension or termination of your participation in this trial. You must maintain all required research records and recognize the IRB is authorized to inspect these records.

IRB approval is valid for a maximum period of one year with continuing review by the IRB

required at least annually in order to maintain approval status. You may not enter subjects on the study before IRB approval or if IRB approval expires. In the latter case, you must immediately contact the IRB to obtain permission to continue subjects on the trial. You must submit a Continuing Review Form sufficiently (one to two months) prior to your study expiration date to permit IRB review before the expiration date.

You must inform the IRB of any unanticipated adverse event or injury no later than ten (10) business days following the time it becomes known that a subject suffered an adverse event/injury. To report external or internal adverse events to the IRB, you must complete and submit the Reportable Event forms in iStar. Furthermore, you must inform the IRB immediately of any significant negative change in the risk/benefit relationship of the research as originally presented in the protocol and approved by the IRB.

INFORMED CONSENT

The request for a WAIVER OF WRITTEN INFORMED CONSENT consistent with 45 CFR 46.117(c) has been approved as iStar #24.4 adequately documents that the research presents no more than minimal risk of harm to the subjects and involves no procedures for which written consent is normally required outside of the research context.

You must use the Verbal Script dated 10/12/2017 approved by the IRB for obtaining informed consent.
Refer to the strikethrough copy of the Verbal Script dated 10/12/2017 for modifications made by the IRB. If you accept the IRB modifications, you may use this Verbal Script for recruitment. If you do not accept the changes or additional changes need to be made, you must make these changes on the clean copy provided by the IRB. All additions and deletions must be identified using the Track Changes feature and the version date must be updated.

The IRB-approved information sheet is located under the “Documents” tab in the iStar study. This is the APPROVED document. You must use a copy of the approved Verbal Script when consenting study participants.

Informed consent must be obtained by the investigator or person authorized to obtain informed consent from all research subjects or their legally authorized representatives. You must ensure that all project personnel involved in the process of consent/assent are trained properly and are fully aware of their responsibilities relative to the obtainment of informed consent/assent according to the IRB guidelines and applicable federal regulations.

HIPAA AUTHORIZATION

The HIPAA Privacy Rule will not apply to this research. The investigator certifies that he/she is not accessing, using or obtaining protected (i.e., identifiable) health information held by; a) a health care provider (e.g., physician or other health care practitioner, hospital, clinic, nursing home); b) health plan (e.g., group health plan, insurance company, HMO); or c) health care clearinghouse (e.g., billing service) that is governed by the HIPAA privacy federal regulations.

Attachments: HS-17-00811_VerbalConsent_IRBAEdits_10-12-2017.docx

Approved Documents: view

This is an auto-generated email. Please do not respond directly to this message using the "reply" address. A response sent in this manner cannot be answered. If you have further questions, please contact your IRB Administrator or IRB/CCI office.

The contents of this email are confidential and intended for the specified recipients only. If you have received this email in error, please notify istar@usc.edu and delete this message.
