# Supplementary material for: Monitoring Twitter Conversations for Targeted Recruitment in Cancer Trials in Los Angeles County: Protocol for a Mixed-Methods Pilot Study
Source: JMIR Res Protoc. 2018 Sep 25;7(9):e177. doi: 10.2196/resprot.9762 (PMC6231794; doi:10.2196/resprot.9762)
Supplement: Multimedia Appendix 4 [file resprot_v7i9e177_app4.pdf]

Locations provided in users' profile descriptions that will be used to identify Twitter users in LA County. Based on data from Symplur.com.

Boolean[a] location code categories

Agoura Hills, Alhambra, Arcadia, Artesia, Avalon, Azusa, Baldwin Park, Bell Gardens, Bellflower, Beverly Hills, Bradbury, Burbank, Calabasas, Carson, Cerritos, City of Angels, Claremont, Commerce, Compton, Covina, Cudahy, Culver City, Diamond Bar, Downey, DTLA, Duarte, El Monte, El Segundo, Gardena, Glendale, Glendora, Hawaiian Gardens, Hawthorne, Hermosa Beach, Hidden Hills, Hollywood, Hollywood, Huntington Park, Industry, Inglewood, Irwindale, La Cañada Flintridge, La Habra Heights, La Mirada, La Puente, La Verne, Lakewood, Lancaster, Lawndale, Lomita, Long Beach, Los Angeles, Lynwood, Malibu, Manhattan Beach, Maywood, Monrovia, Montebello, Monterey Park, Norwalk, Palmdale, Palos Verdes Estates, Paramount, Pasadena, Pico Rivera, Pomona, Rancho Palos Verdes, Redondo Beach, Rolling Hills, Rolling Hills Estates, Rosemead, San Dimas, San Fernando, San Gabriel, San Marino, Santa Clarita, Santa Fe Springs, Santa Monica, Sierra Madre, Signal Hill, South El Monte, South Gate, South Pasadena, Temple City, The Westside, Torrance, Venice, Vernon, Walnut, West Covina, Westlake Village, Westwood, Whittier, Angelino Heights, Arleta, Arlington Heights, Atwater Village, Baldwin Hills, Baldwin Village, Baldwin Vista, Beachwood Canyon, Bel Air, Benedict Canyon, Berkeley Square, Beverly Crest, Beverly Glen, Beverly Grove, Beverly Park, Beverlywood, Boyle Heights, Brentwood, Bunker Hill, Cahuenga Pass, Canoga Park, Canterbury Knolls, Carthay, Castle Heights, Century City, Chatsworth, Chesterfield Square, Cheviot Hills, Crenshaw, Crenshaw, Crestwood Hills, Cypress Park, Del Rey, Eagle Rock, Echo Park, Edendale, El Sereno, Elysian Heights, Elysian Park, Elysian Valley, Encino, Exposition Park, Faircrest Heights, Fairfax, Franklin Hills, Garvanza, Glassell Park, Gramercy Park, Granada Hills, Griffith Park, Hancock Park, Harbor City, Harbor Gateway, Harvard Heights, Harvard Park, Highland Park, Holmby Hills, Hyde Park, Jefferson Park, Kinney Heights, Koreatown, Lafayette Square, Lake Balboa, Lake View Terrace, Larchmont, Laurel Canyon, Leimert Park, Lincoln Heights, Los Feliz, Manchester Square, Mandeville Canyon, Mar Vista, Melrose Hill, Miracle Mile, Mission Hills, Montecito Heights, Monterey Hills, Nichols Canyon, North University Park, Northridge, Pacific Palisades, Pacoima, Panorama City, Park La Brea, Picfair Village, Pico Robertson, Playa del Rey, Playa Vista, Porter Ranch, Rancho Park, Reseda, Reynier Village, Rose Hills, Rustic Canyon, San Pedro, Sawtelle, Sherman Oaks, Silver Lake, Solano Canyon, South Robertson, Spaulding Square, Studio City, Sunland, Sunset Junction, Sylmar, Tarzana, Terminal Island, Toluca Lake, Tujunga, Valley Glen, Valley Village, Van Nuys, Vermont Knolls, Vermont Square, Vermont Vista, Victor Heights, Victoria Park, Warner Center, Watts, West Adams, Westdale, Western Heights, Westlake, Westside Village, Westwood, Whitley Heights, Wilmington, Winnetka, Woodland Hills, Yucca Corridor

**Regex[b] location code categories**
Bell, LA, LAX, PCH, SGV, L.A.X., L.A., k-town

[a] The Boolean location code matches any of the cities listed above in the bio-location field associated with a Twitter user (without regards to case).

[b] The Regex location code matches any of the exact free-standing strings of text listed above in the bio-location field associated with a twitter user, including specified punctuation, if any (without regards to case, or location within the bio-location field).
