# Supplementary material for: Monitoring Twitter Conversations for Targeted Recruitment in Cancer Trials in Los Angeles County: Protocol for a Mixed-Methods Pilot Study
Source: JMIR Res Protoc. 2018 Sep 25;7(9):e177. doi: 10.2196/resprot.9762 (PMC6231794; doi:10.2196/resprot.9762)
Supplement: Multimedia Appendix 5 [file resprot_v7i9e177_app5.pdf]

Keywords and hashtags that will be used for monitoring Twitter user conversations in LA County and for identifying potential clinical trial participants for outreach. Based on data from Symplur.com.

Cancer disease category	Keywords	Hashtags
Breast cancer	"Breast cancer"	#advancedbc (advanced breast cancer)<br>#bccww (breast cancer chat worldwide)<br>#bcmets (breast cancer metastatic)<br>#bcpea (breast cancer patient education action)<br>#bcsm (breast cancer social media)<br>#bctalk (breast cancer talk)<br>#bebrcaware (be BRCA mutation aware)<br>#brca (BRCA mutation)<br>#brca1 (BRCA1 gene)<br>#brca2 (BRCA2 gene)<br>#brcamutation<br>#breastcancer<br>#breastcancerawareness<br>#malebreastcancer<br>#mammogram<br>#mbcsm (metastatic breast cancer social media)<br>#metastaticbc (metastatic breast cancer)<br>#metastaticbreastcancer<br>#pinkribbon<br>#thinkpink<br>#thisismbc (this is metastatic breast cancer)<br>#tnbc (triple negative breast cancer)<br>#trackmbc (track metastatic breast cancer)
Colon cancer	"Colon cancer"<br>"Colorectal cancer"<br>"Bowel cancer"	#bowelcancer<br>#cancercolon<br>#coloncancer<br>#colonoscopy<br>#colorectal<br>#colorectalcancer<br>#crcsm (colorectal cancer social media)<br>#rectalcancer<br>#scopescope
Kidney cancer	"Kidney cancer"<br>Renal (for Renal Cancer and Renal Cell Carcinoma)	#kcsm (kidney cancer social media)<br>#kidneycancer<br>#renalcellcarcinoma
Lymphoma	Lymphoma<br>Hodgkins<br>Hodgkin's<br>Hodgkin	#bloodcancer<br>#bloodcancerawarenessmonth<br>#dlbcl (diffuse large b-cell lymphoma)<br>#hodgkin

	Bloodcancer<br>“Blood cancer”	#hodgkins<br>#lymphoma<br>#lymsm (lymphoma social media)<br>#lyphoma (frequent misspelling of lymphoma)<br>#nonhodgkinslymphoma<br>#edforumchat (annual North American Educational Forum on lymphoma)
Non-small cell lung cancer	“Lung cancer”	#433aday (433 a day; refers to number of people who lose their life to lung cancer every day)<br>#beatlungcancer<br>#changelc (change lung cancer)<br>#chatnsclc (chat non-small cell lung cancer)<br>#endlungcancer<br>#freetobreathe<br>#lccaregiver (lung cancer caregiver)<br>#lcsm (lung cancer social media)<br>#ldct (low dose computed tomograph; lung cancer screening for high risk patients)<br>#lungcancer<br>#lungcancerawareness<br>#lungcancerscreening<br>#nsclc (non-small cell lung cancer)<br>#talklungcancer
Prostate cancer	“Prostate” and word filter “cancer”	#movember (Movember is an annual event involving the growing of moustaches during the month of November to raise awareness of men's health issues, such as prostate cancer, testicular cancer, and men's suicide)<br>#oneineight (one in eight; refers to the statistic that one in eight men will be diagnosed with prostate cancer)<br>#pcsm (prostate cancer social media)<br>#prostatecancer<br>#prostatecancerawareness<br>#prostatecare<br>#prostatechat<br>#psatest (prostate-specific antigen test; refers to screening for prostate cancer)
