# Supplementary material for: Monitoring Twitter Conversations for Targeted Recruitment in Cancer Trials in Los Angeles County: Protocol for a Mixed-Methods Pilot Study
Source: JMIR Res Protoc. 2018 Sep 25;7(9):e177. doi: 10.2196/resprot.9762 (PMC6231794; doi:10.2196/resprot.9762)
Supplement: Multimedia Appendix 6 [file resprot_v7i9e177_app6.pdf]

Consent and prescreening questionnaire for prospective study participants

(i.e., those targeted Twitter users who contacted the study team in response to the social media outreach)

Prospective participant survey consent (oral, over the phone)

Are you 18 or older?

- Yes (proceed)
- No (We are sorry but participants must be 18 or older to participate. We appreciate your interest.)

Purpose of the study: We are studying the acceptability and effectiveness of social media listening and recruitment on Twitter to enhance enrollment for cancer-related clinical trials. Your participation in this 5-min survey will help us understand who we were able to reach with these messages. We will ask you demographic information, such as your age, gender, education, and your opinion about the use of Twitter, in particular listening to Twitter user conversations, for cancer clinical trial recruitment.

Voluntary participation: Participating in this survey is entirely voluntary. You can withdraw from the survey or skip questions at any time without negative consequences. The information you provide will be confidential and only used for the purpose of this research project. Your information will not be shared with third parties.

Cost: There is no cost to you for taking part in this study and completing this survey.

Compensation: There is no compensation for taking part in this study and completing this survey.

If you have further questions, feel free to contact the study team at the University of Southern California. Principal investigator: Thomas Buchannan (buchanan@usc.edu). If you have any questions about your rights as a research participant, or want to talk to someone independent of the research team, you may contact the Institutional Review Board Office at 323-223-2340 or email at irb@usc.edu).

Do you consent?

- Agree
- Don't agree.

USC Children's Hospital Los Angeles LAC+USC iStar

Proposal #HS-17-00811

University of Southern California Health Sciences Campus
Institutional Review Board
LAC+USC Medical Center, General Hospital Suite 4700
1200 North State Street, Los Angeles, CA 90033
(323) 223-2340 phone
(323) 224-8389 fax
irb@usc.edu

Date: Nov 01, 2017, 11:46am

To: Thomas Buchanan, M.D.
ENDOCRINOLOGY AND DIABETES
Daisy Sosa, BA
Data Manager
CLINICAL INVESTIGATIONS SUPPORT OFFICE (CISO)

From: Health Sciences Institutional Review Board
General Hospital Suite 4700
1200 North State Street
Los Angeles, CA 90033
(323) 223-2340

TITLE OF PROPOSAL:
0S-17-7: The use of social media listening for targeted recruitment of Twitter users in LA County in cancer trials compared to historic recruitment data: A mixed-methods study
Amendment: HS-17-00811-AM001 (0S-17-7 Protocol version 4 dated 1Nov2017)

Action Date: **11/1/2017** Action Taken: **Approve**

Committee: Institutional Review Board Chairman

Note: Your IRB submission received on 11/01/2017 was reviewed by Dr. Linda Sher on 11/01/2017.

The proposed changes qualify for expedited review according to 45CFR46.110(b)(2) minor changes in previously approved research during the period (of one year or less) for which approval is authorized.

The proposed changes were APPROVED.

The revised iStar Application dated 11/01/2017 was APPROVED.

The revised Protocol, dated 11/01/2017 was APPROVED.

The revised Questionnaire, dated 11/01/2017 was APPROVED.

Attachments:

Approved ICs and HIPAA forms: view

This is an auto-generated email. Please do not respond directly to this message using the "reply" address. A response sent in this manner cannot be answered. If you have further questions, please contact your IRB Administrator or IRB/CCI office.

The contents of this email are confidential and intended for the specified recipients only. If you have received this email in error, please notify istar@usc.edu and delete this message.

Prospective participant data collection sheet

1. Name of prospective participant
   a. First name
   b. Last name

2. What's your Twitter handle?

   a. ________________________
   b. Prefer not to disclose

3. What's your email?

4. What's your date of birth?

5. Are you…?
   - Female
   - Male
   - Other
   - Prefer not to disclose

6. How do you describe yourself? (check all that apply)
   - African American / Black
   - American Indian / Alaska Native
   - Asian / Pacific Islander
   - Hispanic
   - Middle Eastern
   - White
   - Other
   - Prefer not to disclose

7. What is the highest degree or level of school you have completed?
   - No schooling completed
   - Nursery school to 8th grade
   - Some high school, no diploma
   - High school graduate, diploma or the equivalent (for example: GED)
   - Some college credit, no degree
   - Trade/technical/vocational training
   - Associate degree
   - Bachelor's degree
   - Master's degree
   - Professional degree
   - Doctorate degree
   - Prefer not to disclose

8. Did you respond to the social media recruitment message because you would like to learn more about a clinical trial?
    a. Yes
    b. No

9. Are you gathering information for yourself or on behalf of someone else?
    a. For myself
    b. On behalf of someone else

10. How helpful would you say is it when research institutions reach out directly to potential clinical trial participants via Twitter?
    a. Very helpful
    b. Helpful
    c. Somewhat helpful
    d. Not helpful at all
    e. Don't know

11. How concerned are you about researchers and research institutions monitoring Twitter conversations to identify and contact potential study participants for clinical trials?
    a. Very concerned
    b. Somewhat concerned
    c. Not too concerned
    d. Not concerned at all
    e. Don't know

12. When were you diagnosed with cancer?

13. Do you have active cancer? (Only if yes triage to USC Norris team)
    a. Is it visible on scans (CT, MRI)?
    b. Are you on active treatment?

14. Are you able to do activities of daily life independently? (e.g., eating, drinking, bathing)

15. Do you have a copy of your medical records?

16. Are you currently a patient at
    a. USC Norris Comprehensive Cancer Center, or
    b. USC Keck Medical Center, or
    c. LA County USC?

17. Any other comments you want to share (free text)
