# Supplementary material for: Monitoring Twitter Conversations for Targeted Recruitment in Cancer Trials in Los Angeles County: Protocol for a Mixed-Methods Pilot Study
Source: JMIR Res Protoc. 2018 Sep 25;7(9):e177. doi: 10.2196/resprot.9762 (PMC6231794; doi:10.2196/resprot.9762)
Supplement: Multimedia Appendix 7 [file resprot_v7i9e177_app7.pdf]

SUMMARY STATEMENT
(Privileged Communication)

PROGRAM CONTACT:
Patricia Jones
240-292-4851
jonespl@mail.nih.gov

Release Date: 04/08/2016 11:14 AM

Revised Date:

Application Number: 1 U54 TR001350-01A1

Principal Investigators (Listed Alphabetically):

BUCHANAN, THOMAS A (Contact)
KIPKE, MICHELE D.

Applicant Organization: UNIVERSITY OF SOUTHERN CALIFORNIA

Review Group:	ZTR1 CI-8 (01)<br>National Center for Advancing Translational Sciences Special Emphasis Panel<br>CTSA Review
Meeting Date:	02/10/2016
Council:	MAY 2016
Requested Start:	07/01/2016
RFA/PA:	PAR15-304
PCC:	CRT35
Project Title:	Southern California Clinical and Translational Science Institute
SRG Action:	Impact Score:20
Next Steps:	Visit http://grants.nih.gov/grants/next_steps.htm
Human Subjects:	30-Human subjects involved - Certified, no SRG concerns
Animal Subjects:	30-Vertebrate animals involved - no SRG concerns noted
Gender:	1A-Both genders, scientifically acceptable
Minority:	1A-Minorities and non-minorities, scientifically acceptable
Children:	1A-Both Children and Adults, scientifically acceptable<br>Clinical Research - not NIH-defined Phase III Trial

[redacted]

ADMINISTRATIVE BUDGET NOTE: The budget shown is the requested budget and has not been adjusted to reflect any recommendations made by reviewers. If an award is planned, the costs will be calculated by Institute grants management staff based on the recommendations outlined below in the COMMITTEE BUDGET RECOMMENDATIONS section.

1U54TR001350-01A1 PI Buchanan, Thomas A.

RESUME AND SUMMARY OF DISCUSSION: This is a resubmission application for the Clinical and Translational Science Award (U54) from The University of Southern California entitled "Southern California Clinical and Translational Science Institute."

[redacted]

Strengths of the proposed CTSA program include the clear organizational structure and the highly-qualified collaborative leadership; the focus on community partnerships, health disparities, and health communication; well-developed clinical informatics; an emphasis on team science; a high impact Pilot Funding Program; strong institutional support for translational science; an impressive scientific environment; and the components of Informatics, Community and Collaboration, Translational Endeavors, Research Methods, Hub Research Capacity, Network Capacity, and the optional Digital Innovation and Child Health-Research Acceleration through Multisite Planning (CHAMP) programs. Enthusiasm for the UL1 components is somewhat reduced by the apparent advisory, as opposed to decision-making, role of the community partners and by the fact that most CTSI activities occur at USC. Plans for the Institutional Career Development Core (KL2) are strong, although it is noted that the mentor pool is somewhat narrow relative to the translational spectrum and that some of the newly-recruited mentors are relatively junior. In this resubmission application, the applicants have been responsive to the criticism of reviewers from the previous cycle and have made appropriate changes. This CTSA has tremendous potential to have very high impact.

The UL1 components have numerous strengths. The Multiple Principal Investigators (MPIs) are experienced, possess strong translational science backgrounds, and are well suited to develop this CTSA hub into a high performance network. Additional strengths include the well-defined governance structure; broad representation of investigators from multiple fields; a substantial emphasis on implementation of team science; a well-developed Informatics Program; a high impact Pilot Funding Program with specific metrics of success; and exceptional institutional commitment to translational research. The SC CTSI builds on its location in terms of the unique underserved urban population in the surrounding area, and there is a focus on community partnerships, health disparities, and health communication throughout the application. There is a well-developed program for integrating special populations, including children and the elderly; an innovative plan to use digital approaches for outreach; a specific accrual monitoring plan; and well-defined strategies to increase efficiency in clinical trials. There are some concerns noted in that it not clear to what extent community partners have ongoing decision-making, in addition to advisory, responsibilities; there are no Key Personnel from outside academia; and one institution appears to be more involved, as most CTSI activities occur at USC.

Strengths of the Institutional Career Development Core (KL2) include a highly-qualified Director who has had success in the administration of this program; a broad range of experienced mentors from a variety of scientific disciplines and clinical research interests; well-described and appropriate recruitment plans for identifying early-career Scholars; a high-quality pool of potential participants whose numbers are appropriate to the program size; protected time for Scholars; and impressive institutional support to expand the cohort of Scholars. The learning environment and tools are excellent, and the KL2 Program leverages CTSA strengths in Community Engagement, clinical informatics, and team science to emphasize the development of clinical investigators with career interests focused on diverse patient populations. In addition, the timeline for the program has appropriately been extended from two to three years to allow additional time for Scholar development, and productivity of recent Scholars has been excellent in terms of by publications and success in obtaining grants. It is noted that the mentor pool is somewhat narrow relative to the translational spectrum and that some of the newly-recruited mentors are relatively junior and will require training in mentorship.

Overall, the application received an Impact/Priority Score of 20; the committee recommended the budget as requested.

DESCRIPTION (provided by applicant): The Southern California Clinical and Translational Science Institute (SC CTSI) is a multi-disciplinary research institute located at the University of Southern California and Children's Hospital Los Angeles. In partnership with the Los Angeles County Department of Health Services and broad-based community health organizations throughout Los Angeles, the SC CTSI seeks to be a leader in engaging diverse communities and special populations in clinical and translational research to improve their health. It is led by a team of faculty and staff members who have deep experience in developing and supporting clinical and translational research. They propose to address issues of health across the lifespan, as well as challenges in clinical and translational research, by pursuing six specific aims: (1) Integration: engage diverse communities and special populations, their care providers and researchers to establish clinical research priorities; identify barriers to research; and develop, demonstrate and disseminate innovative approaches to assure fully partnered clinical research across communities and the lifespan; (2) Collaboration: continue our successful program of team building in support of research that addresses health needs and research challenges; complement it by identifying successful elements and best practices and developing methods to support distributed ("virtual") teams; (3) Workforce Development: expand workforce capacity and capabilities by training researchers, staff, and clinical and community partners in team-based research with special emphasis on diverse populations; (4) Methods and Processes: streamline processes to support safe, efficient and high quality local and multicenter clinical research, in particular in diverse clinical and community settings; (5) Informatics: employ and develop innovative informatics solutions to enhance clinical and translational research, integrate research and clinical care in pursuit of learning healthcare systems, and integrate research and community health in pursuit of a culture of health; (6) CTSA Hub: participate in CTSA network activities, conduct multi-site studies, adopt successful models from peers, and develop and disseminate innovative approaches. We bring to the network special strengths in community engagement, diversity, digital innovation, and regulatory science. Success in achieving our aims will create new solutions for health problems affecting some of the fasting growing and most vulnerable special populations in America, define new approaches to research with diverse communities, and contribute our deep experience and expertise to the national CTSA network.

PUBLIC HEALTH RELEVANCE (provided by applicant): This application is for support of the Southern California Clinical and Translational Science Institute (SC CTSI). The SC CTSI is a multi-disciplinary institute housed at the University of Southern California and Children's Hospital Los Angeles. In partnership with the Los Angeles County Department of Health Services and broad based community health organizations in Los Angeles, the SC CTSI seeks to develop and support multidisciplinary clinical and translational research that will improve the health of diverse communities and address challenges in the conduct of clinical research with those communities.

CRITIQUE

CTSA UL1 Components: Administrative Core, Informatics, Community and Collaboration, Translational Endeavors, Research Methods, Hub Research Capacity, Network Capacity, and Optional Functions

Critique 1

Significance: 2

Investigator(s): 2

Innovation: 2

Approach: 2

Environment: 1

Overall Impact

The Southern California Clinical and Translational Science Institute (SC CTSI) has a unique focus on community partnerships, health disparities and health communication with the USC School of Cinematic Arts and the Hollywood Health and Society Program at the USC Annenberg Center. They are focused on health disparities in urban LA and have created an interdisciplinary Center for Health Systems Innovation (CHS) to promote a learning health system with a goal of better engaging health systems and clinicians. Components are well focused on their distinctive setting, patient populations and unique strengths at USC. This is a strong application with well-developed programs, excellent national collaborations, and novel approaches to patient engagement, particularly using digital technologies. The applicants have been sensitive to the prior review and have enhanced an already strong team to better integrate clinical trials and research tools across the clinical partners, and to improve engagement of diverse populations including children and the elderly. This CTSA brings novel strengths to the CTSA network with outreach strategies and digital technologies that complement clinical informatics strategies nationally.

Significance

Strengths

- The applicants carefully address concerns from a prior review regarding governance, reporting relationships of the CTSI and succession planning. The CTSA Principal Investigators, Thomas Buchanan, M.D. and Michele Kipke, Ph.D. report to the Provost, Michael Quick, Ph.D., to ensure the CTSI has broad influence.

- The CTSI investigative team has been broadened with additional M.D.s and Ph.D.s. The rationale for inclusion of various team members is strong and the Multiple Principal Investigator (MPI) team members have complementary skills.
- The governance structure is well defined and includes internal and external committees with well-defined functions and schedules.
- There are specific plans for collecting evaluation metrics including a well-described Data Nexus automatic tracking and reporting system. The system is now functional for consultation services but has not been fully deployed.
- The applicants clearly describe a strong External Advisory Committee (EAC) and give specific examples of changes made in response to EAC recommendations (e.g., eliminate a partner, expanded collaboration with the University of California Los Angeles (UCLA) CTSA hub).
- The applicants have a well-developed pilot program and provide specific metrics of success. They have also collaborated on metrics development for the national CTSA network.
- There is a well-developed program for integrating special populations, including children and the elderly. The applicants have been responsive to prior concerns insufficient breadth in their population focus.
- Specific plans are presented related to research coordinators in terms of their training and availability to support trials. The applicants propose virtual teams, have written a book on the subject, and offer workshops on the use of technology to improve team interaction, along with workshops on other key elements of team science.
- A specific accrual monitoring plan is presented. OnCore is being implemented on a system-wide basis, and there are Research Ambassadors to support its use. OnCore will be linked to the Data Nexus system to allow comprehensive tracking.
- Specific plans for Liaison to Trial Innovation Centers (LTICS) and Liaison to Recruitment Innovation Centers (LRICS) are described including hiring a new Clinical Research Director, building on prior strengths such as strong recruitment of underrepresented minorities (URM), Institutional Review Board (IRB) reliance agreements, and progress in clinical informatics.

Weaknesses

- The applicant notes they expect Data Nexus to be more functional by the second quarter of 2016. This is an interesting system with great potential, but it is not yet integrated with other clinical research tools. It would have been helpful to have a better description of the specific steps that are being taken to make the tool more functional.

Investigator(s)

Strengths

- The SC CTSI investigators are well suited to transform the CTSA program into a high-performance network. Dr. Thomas Buchanan has a strong translational research background in Latino health (gestational and Type 2 diabetes, T2D). Dr. Michele D. Kipke has conducted outstanding translational research related to Human Immunodeficiency Virus (HIV) and Autism Spectrum Disorders (ASD).

- The Core Directors and co-Directors are well qualified. Daniella Meeker, Ph.D. is an investigator with the patient-centered Scalable National Network for Effectiveness Research (pSCANNER) and the National Patient-Centered Clinical Research Network (PCORnet) and leads the data harmonization approaches, which are important for translational research.
- The overall team is experienced, diverse, and complementary; this provides a high likelihood for success in the development, demonstration, and dissemination of approaches for treatment and prevention. The team incorporates expertise in a range of methods, age cohorts, research venues, and diseases.
- The Clinical Research Support team incudes experts in biostatistics and multisite study support.
- Hub investigators are all successful at the national and international level. Investigators with expertise on brain development and stroke have been added.
- The applicants are invested in improving and identifying best practices. They have developed a Clinical Research Advisory Group (CRAG) to identify high priority challenges to clinical research.
- The applicants have implemented a learning health system program (Center for Health System Innovation).
- There are well-defined strategies to increase efficiency in clinical trials including Research Navigators, Biostatistics, Epidemiology and Research Design (BERD), recruitment specialists, feasibility reviews, and OnCore implementation.
- The applicants are clearly invested in innovation in clinical and translational research (CTR) and have proposed innovative strategies for recruiting parents and children and for the use of social media.
- The applicants have been sensitive to prior concerns about being appropriately resourced. New staff has been added: of 22 total staff members, 21 are now hired and the one remaining “to-be-named” is for a new initiative with geocoded data integration.

Weaknesses

- The applicants note they will use feedback from the CRAG, researcher surveys, and the Data Nexus system to streamline clinical research processes. The example provided is related to implementing a tracker for IRB submissions. This is an issue that should already have been addressed within the CTSA.

Innovation

Strengths

- The SC CTSI clearly delineates projects that are being conducted in collaboration with other CTSAs focused on Community Engagement and return-on-investment (ROI) methodology. ROI methodology looks at the extent to which studies impact outcomes. Contributions in these areas could have a broad effect on CTR.
- There is an additional collaborative focus with PCORnet. Dr. Meeker is an investigator with pSCANNER, which involves seven other sites with CTSA grants.
- The applicants focus extensively on vulnerable populations and have developed a strong plan to include them.

- The use of digital approaches for outreach is innovative.
- The applicants propose interactions with colleagues in Journalism (USC Annenberg) to expand recruitment and information dissemination.

Weaknesses

- The applicants are focused on their own community setting which is a more narrow focus. However, they are applying innovative approaches to their setting.

Approach

Strengths

- The Informatics section is well developed. The applicants have implemented underlying data warehouse structures at the Keck School of Medicine and LA Children's Hospital.
- There is a range of information technology (IT) tools, including i2b2, PopMedNet, and eIRB.
- The applicants can query electronic health records (EHR) from their clinical partners and from pSCANNER. The ability for query execution is well documented, and participation with pSCANNER is an asset
- The applicants have query support and an honest broker system with their partners. Tools are user friendly.
- Standard terminologies are used and quality management plans are well defined.
- Specific plans for geocoding and spatial modeling are provided along with information about social determinants of health.
- Body Mass Index (BMI) engagement strategies for health systems to identify issues and work with those issues as clinical and research priorities are well documented.
- A table of research IT objectives, strategies and metrics for measuring outcomes is provided.
- Four workforce development initiatives are described including IT short courses for engineers, training for researchers in digital innovation, a Research Coordinator certificate and a Core Competency short course for clinical investigators.
- Importantly the applicants link their informatics program to their focus on Community Engagement, continuously learning health systems, children, and health disparities.
- The Community Engagement Program has strong leadership and the team has a strong portfolio of 24 pilot projects.
- The applicants propose a focus on a geospatial data repository (Community Vital Signs) to help develop community dashboards and address barriers to research participation among diverse populations.
- The applicants use tools from other CTSA hubs for Community Engagement and information dissemination; they have customized the approaches for the east LA population. The applicants indicate commitment to using common metrics and serve as the coordinating center for measuring Community Engagement with other CTSAs.
- The applicants describe an interest in expanding training to community clinicians through boot camps, short courses, and virtual journal clubs.

- Some team members have limited research experience and their specific role within the CTSI is not clearly outlined in their personal statement.
- Some team members seem to have primarily administrative roles, or they have no active support aside from the previous CTSA; this suggests a somewhat passive role within the Center.
- Some faculty who will serve as instructors have no funded research and appear to have primarily clinical expertise. It is not clear how they were selected for such a key role.
- The Provost's presence on the IOB could unduly influence the overall direction of the Board.
- The Department of Preventive Medicine seems to be overrepresented.

Innovation

Strengths

- Investigators on the application had prior research experience with both children and older adults and there are plans to include both populations.
- Several ongoing and new initiatives are mentioned to innovate clinical research. Eight different ongoing collaborations with other hubs are mentioned.
- There is a plan to partner with the entertainment industry to promote health-related messages.
- There is a plan to establish collaborations with the Los Angeles County Department of Health Services.
- The CTSI is involved in a number of services aimed at facilitating community-based trials.
- The CTSI will facilitate the development of a new course on clinical research for IT personnel to improve the integration of IT and research.
- There are close ties with the urban population of Los Angeles, which consists of highly diverse subgroups.

Weaknesses

- It is not clear to what extent community partners have ongoing and integrated decision-making responsibilities, as they seem to be mostly advisory.
- It appears that studies will be primarily focused on the urban population of Los Angeles.
- It is not clear if collaborations with other hubs were specifically facilitated by the CTSI, or if the CTSI was a bystander.
- It is not clear if some of the new online training opportunities will be freely available to investigators from other hubs or to others outside USC, though there is mention that current opportunities are shared with other hubs.
- Description of proposed activities does not provide in-depth details or discussions of barriers and solutions.

Approach

Strengths

- There are well-defined working groups with specific tasks. For example, the Digital Innovation and Communication Group will develop and evaluate several innovations to overcome barriers to participation in research.
- The CTSI has been influential in efforts to reward team science and clinical research participation by clinicians by updating promotion and tenure criteria.
- There are multiple plans mentioned to integrate databases and utilize mobile technologies. A number of activities are already in progress, such as developing real-time tracking systems for clinical trials.
- Recent technologies, such as the use of avatars as Research Ambassadors, will be leveraged to enhance research activities and identify community health priorities.
- The USC CTSI is working with the UCLA CTSA to create a federated city-wide data warehouse for research.
- Clinical Research Support offices have already been centralized between USC and CHLA.
- The CTSI will launch online training on regulatory aspects of clinical trials to promote Good Clinical Practice (GCP).
- There is a plan for the Clinical Research Working group to develop Quality and Efficiency metrics; it is not clear what metrics have been used until now and if benchmarks have been met.
- Data from communities, including geospatial data, will be integrated with EHR to incorporate social determinants of health through Community Vital Signs.
- The data quality campaigns to improve EHR are well described, innovative, and needed.

Weaknesses

- There have been a number of university-wide changes to facilitate research, though it is not always clear to what extent these changes would have happened without the CTSI.
- There is limited information on what key performance metrics and formative evaluations are used throughout the CTSI components and integrated into a systematic global assessment plan. It is also not completely clear who will be responsible for the assessments, and how the outcomes will be used for improvement.
- Although there is mentioned that the USC hub has joined PCORnet, there is a limited description of how this will be used to facilitate research.
- There is mention that the CTSA is ready to participate in multicenter clinical trials; however, there is no description of anticipated barriers.
- It is not clear if other clinicians will receive credits specific to their profession and other than Continuing Medical Education (CME) credits, and will have an equal opportunity to be trained in clinical research through the CTSI.
- It is not clear how active or powerful the role of community partners will be aside from a limited advisory capacity. There are only two community leaders part of the IOB. representing over 60 community organizations.
- It is not clear how much of Community Vital Signs has been built, as the resource section mentions that it will be built, but in the research plan it appears to already be underway.

Significance: 2

Investigator(s): 3

Innovation: 2

Approach: 3

Environment: 2

Overall Impact

This grant application is from two partner institutions, the University of Southern California and Children's Hospital Los Angeles. The MPIs have significant expertise in leading the CTSI and have assembled a diverse team. However, a limitation is that some USC schools appear to have no or limited representation among Key Personnel, whereas some departments in the Medical School are overrepresented. There is a plan to strengthen links to the county health Department, although partnerships with the private sector are less obvious. The hub will utilize the latest technologies to advance research and is working towards greater data integration, with the integration focused on county-level data warehousing. The informatics structure is well defined and strong, with definite plans for advancement. The planned use of technology is innovative. The organizational structure is well described with clear roles of specific groups, though individual roles of some Key Personnel are less clear. The structure includes a biostatistics service that is well utilized, though methodology training offered to the large pool of investigators other than K Scholars seems somewhat limited. K Scholars have been from diverse training backgrounds and have been increasing. Some quality metrics still need to be defined and will include common CTSA metrics. The hub has strong links to the surrounding community, and community members will be part of advisory committees and focus groups, but it is not clear if community members have ongoing decision-making responsibilities. Overall, the hub appears to have a strong ability to continue to evolve, adapt, and expand; anticipated barriers are not always well described. Overall, this is an impressive grant application and the proposed plan has a high potential for sustained impact.

Significance

Strengths

- It appears the CTSI will be well-integrated within the institutions and will serve as a catalyst for research.
- There are defined innovative plans to integrate several databases.
- There are plans to develop more metrics that are specific to the applicant institution and common to the broader CTSI community.
- The integration of community-level data and medical data will provide new opportunities for research.
- This is a productive CTSI, with an expanding KL2 Program.

Weaknesses

- Most of the CTSI activities will occur in one of the two participating institutions (USC).
- The metrics and evaluation of quality and performance do not appear to be part of a well-structured global assessment plan.

- The integration of basic science/laboratory work with clinical efforts is not well described.

Investigator(s)

Strengths

- Dr. Buchanan has extensive CTSI leadership experience.
- Investigators from multiple disciplines are included as Key Personnel.
- The partnership with the Spatial Sciences Institute investigators can facilitate data diversification and expansion, with inclusion of data beyond only clinical.
- CTSI Scholars have been very productive in publishing manuscripts and obtaining funding for testing new research ideas.

Weaknesses

- Some schools aside from the Medical School have no Key Personnel listed. The limited presence and role for the investigators from Children's Hospital may limit opportunities for integration and collaboration across the two institutions.
- The involvement of community leaders in CTSI leadership seems limited and only advisory. In the Internal Oversight Board, there are two representatives for more than 60 communities listed.
- The community members provide information on community health issues and dissemination techniques, but they do not appear to be actively involved in research development/design alongside the investigators.
- There are no research-active community members listed as mentors.
- Some mentors do not appear to have sufficient research experience to be strong mentors.
- Although inclusion of junior investigators is commendable, some investigators do not appear to be sufficiently experienced for their assigned role, and their activities appear to be primarily focused on clinic or teaching rather than research.
- Additionally, some investigators do not appear to have developed independent research careers beyond their prior funding in the CTSI. Some have not produced significant scholarly work. The process of selection for these investigators for additional roles/responsibilities in the current CTSA application is not described.

Innovation

Strengths

- The metrics and benchmarks are well-defined for some components.
- There is a plan to provide more robust study findings dissemination outside University walls by pairing investigators and communities.
- Innovative plans to utilize current technologies have been proposed.
- There are innovative plans to cross-train individuals across multiple fields, including non-traditional types of training.
- The new Digital Scholars Program is innovative.
- The use of social media to listen to communities is innovative.
